# Functional mechanism study of the allelochemical myrigalone A identifies a group of ultrapotent inhibitors of ethylene biosynthesis in plants

**DOI:** 10.1016/j.xplc.2024.100846

**Published:** 2024-03-08

**Authors:** George Heslop-Harrison, Kazumi Nakabayashi, Ana Espinosa-Ruiz, Francesca Robertson, Robert Baines, Christopher R.L. Thompson, Katrin Hermann, David Alabadí, Gerhard Leubner-Metzger, Robin S.B. Williams

**Affiliations:** 1 Centre for Biomedical Sciences, Department of Biological Sciences, Royal Holloway University of London, Egham TW20 0EX, UK; 2 Centre for Plant Molecular Sciences, Department of Biological Sciences, Royal Holloway University of London, Egham TW20 0EX, UK; 3 Instituto de Biología Molecular y Celular de Plantas (CSIC-UPV), 46022 Valencia, Spain; 4 Centre for Life’s Origins and Evolution, Department of Genetics, Evolution and Environment, University College London, London, UK; 5 Syngenta, Crop Protection AG, Stein, Switzerland

**Keywords:** 1-aminocyclopropane-1-carboxylic acid, ACC, ACC oxidase, ACO, allelochemicals, ethylene synthesis inhibitors, structure–activity relationship

## Abstract

Allelochemicals represent a class of natural products released by plants as root, leaf, and fruit exudates that interfere with the growth and survival of neighboring plants. Understanding how allelochemicals function to regulate plant responses may provide valuable new approaches to better control plant function. One such allelochemical, Myrigalone A (MyA) produced by *Myrica gale*, inhibits seed germination and seedling growth through an unknown mechanism. Here, we investigate MyA using the tractable model *Dictyostelium discoideum* and reveal that its activity depends on the conserved homolog of the plant ethylene synthesis protein 1-aminocyclopropane-1-carboxylic acid oxidase (ACO). Furthermore, *in silico* modeling predicts the direct binding of MyA to ACO within the catalytic pocket. In *D*. *discoideum*, ablation of *ACO* mimics the MyA-dependent developmental delay, which is partially restored by exogenous ethylene, and MyA reduces ethylene production. In *Arabidopsis thaliana*, MyA treatment delays seed germination, and this effect is rescued by exogenous ethylene. It also mimics the effect of established ACO inhibitors on root and hypocotyl extension, blocks ethylene-dependent root hair production, and reduces ethylene production. Finally, *in silico* binding analyses identify a range of highly potent ethylene inhibitors that block ethylene-dependent response and reduce ethylene production in *Arabidopsis*. Thus, we demonstrate a molecular mechanism by which the allelochemical MyA reduces ethylene biosynthesis and identify a range of ultrapotent inhibitors of ethylene-regulated responses.

## Graphical abstract

**Figure:**
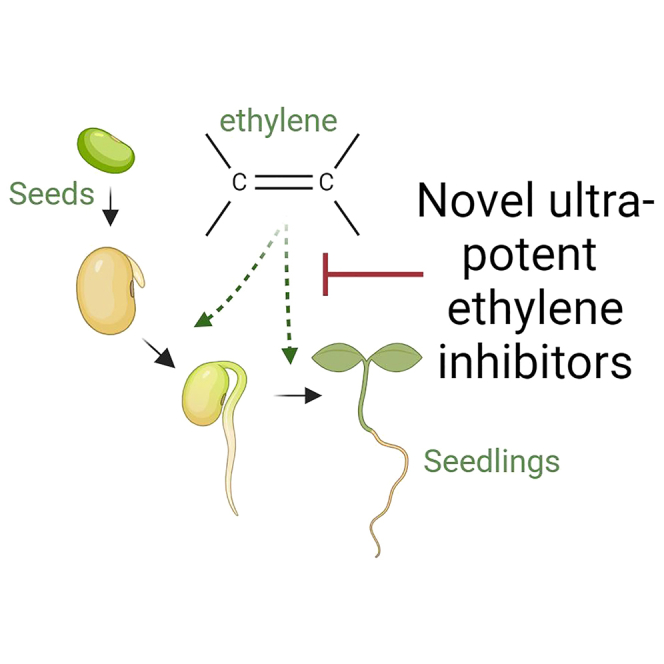

## Teaser

This study reports the investigation of the molecular mechanism of the allelochemical Myrigalone A (MyA) using *Dictyostelium* model system. It provides evidence that MyA targets the ethylene biosynthesis protein ACO, leading to reduced ethylene production, and demonstrates this mechanism in plants. Furthermore, a novel group of ultrapotent ethylene inhibitors effective in plants have been identified through *in silico* modeling of related compounds for the same mechanism.

## Introduction

Developing new approaches for controlling plant growth and development is critical for innovation in agriculture and horticulture, and plant-derived secondary metabolites that show allelochemical phytotoxic effects may provide these approaches (Weston and Duke, 2003). For example, there is a pressing need to develop new herbicides (Busi et al., 2013) to replace those being withdrawn owing to environmental and safety concerns (Heap, 2014) and to provide new effective and sustainable herbicides with unique modes of action and improved toxicological and environmental profiles (Dayan and Duke, 2014). Allelochemicals are typically less harmful to both the environment and humans, often lack the problems associated with the off-target effects of synthetic bioherbicides, and are biodegradable with reduced likelihood of long-term environmental effects (Weston and Duke, 2003).

Myrigalone A (MyA) is a poorly characterized allelochemical derived from fruit and leaf exudates of *Myrica gale* (bog myrtle), a small deciduous shrub native to wetlands in Europe and North America (Skene et al., 2000). MyA is an unusual C-methylated flavonoid that inhibits seed germination and seedling growth in a range of plant species—*Lepidium sativum* (Oracz et al., 2012; Voegele et al., 2012), *Fallopia* × *bohemica*, *Sorghum saccharatum*, and *Sinapis alba* (Popovici et al., 2011). Although its mechanism of action remains unclear, MyA inhibits micropylar endosperm cap weakening and radical/hypocotyl growth, leading to a delay in endosperm rupture and seed germination (Holdsworth et al., 2008; Oracz et al., 2012). Thus, improving our understanding of the molecular mechanism underlying these effects may help to establish a new molecular target and mechanism for the control of plant growth and development.

Here, we used *Dictyostelium discoideum* to further our understanding and identify the molecular target of MyA. This unicellular amoeba is a member of the *Protozoa*, nestled between the plant and animal kingdoms (Eichinger et al., 2005), and contains orthologous proteins and pathways found in both kingdoms, enabling a range of experimental approaches to identify the molecular mechanisms of bioactive compounds in plants (Schaf et al., 2019). *D*. *discoideum* has been used to investigate a range of plant-derived compounds at a molecular level, including curcumin and related compounds (Cocorocchio et al., 2018), medium-chain fatty acids derived from coconut (Chang et al., 2012; Warren et al., 2018, 2020), cannabinoids (Perry et al., 2020; Damstra-Oddy et al., 2021), and multiple plant-derived compounds with bitter or aversive properties (Robery et al., 2013; Cocorocchio et al., 2016). This model organism enables the use of unbiased chemical-genetic screens to identify genes (mutants) that control the sensitivity of cell proliferation to bioactive compounds (Schaf et al., 2019). The theory behind this process is that insertional mutants within a library have adapted to the loss of individual proteins in growth by altered regulation of signaling pathways. Thus, if a mutant has lost a protein targeted by a bioactive compound, it will show at least partial resistance to the inhibitory effect of that compound on cell proliferation. Mutations that result in compound resistance often occur in the target proteins (Roemer and Boone, 2013) and can thus be used to identify specific compound targets. In addition, the *D*. *discoideum* life cycle has distinct unicellular proliferation (growth) and multicellular (developmental) phases, which enable unique approaches to the investigation of bioactive compounds. The switch between these two phases of the life cycle is triggered by starvation, leading to a well-characterized process of multicellular development and formation of a mature fruiting body. This involves an array of proteins and signaling processes that are often distinct from those needed for cell proliferation (Loomis and Shaulsky, 2011) and can be quantified through known expression patterns. Both qualitative and quantitative analyses of the developmental effects of bioactive compounds in this model have been widely used in molecular mechanism studies (McQuade et al., 2013; Ludtmann et al., 2014; Garige and Walters, 2015; Cocorocchio et al., 2018). Finally, isogenic knockout mutants can be rapidly generated with CRISPR technology (Sekine et al., 2018) to investigate the loss of sensitivity to bioactive compounds, which can be re-introduced through expression of endogenous, mutated, or heterologous genes (Ludtmann et al., 2014; Cocorocchio et al., 2016; Perry et al., 2020; Warren et al., 2020; Damstra-Oddy et al., 2021). Thus, *D*. *discoideum* provides an effective model system for the analysis of plant-derived bioactive compounds.

Here, we show that MyA both reduces *D*. *discoideum* unicellular proliferation and delays multicellular development. Through an unbiased chemical-genetic screen, we identify a *D*. *discoideum* mutant that has lost the gene encoding an ortholog of plant 1-aminocyclopropane-1-carboxylic acid oxidase (ACO) and is resistant to the effect of MyA on cell proliferation and development. In plants, ACO catalyzes the final step in ethylene production (Houben and Van de Poel, 2019); it is an important hormone for the regulation of seed germination (Corbineau et al., 2014), plant growth, and senescence (Iqbal et al., 2017). We reveal that development of the *D*. *discoideum Aco*^−^ mutant phenocopies that of wild-type cells treated with MyA, implying that loss of ethylene production triggers a delay in development. Furthermore, this developmental delay can be partially rescued with exogenous ethylene, which also restores developmental marker expression. Both treatment of wild-type cells with MyA and loss of the *Aco* gene result in reduced ethylene production. Molecular modeling suggests the direct binding of MyA within the active-site pocket of both *D*. *discoideum* and plant ACO enzymes. Translation of this mechanism to *Arabidopsis thaliana* shows that MyA blocks seed germination and reduces root and hypocotyl extension in seedlings, effects that are reproduced by established ethylene inhibitors. In addition, MyA blocks ethylene-induced increases in root hair number and length. Finally, through molecular modeling and quantitative structure–activity relationship analysis in *D*. *discoideum* and plant bioassays, a range of highly potent ethylene inhibitors have been identified, providing a new class of compounds for inhibition of ethylene biosynthesis in plants.

## Results

### MyA inhibits *D. discoideum* cell proliferation and delays multicellular development

**Figure 1:**
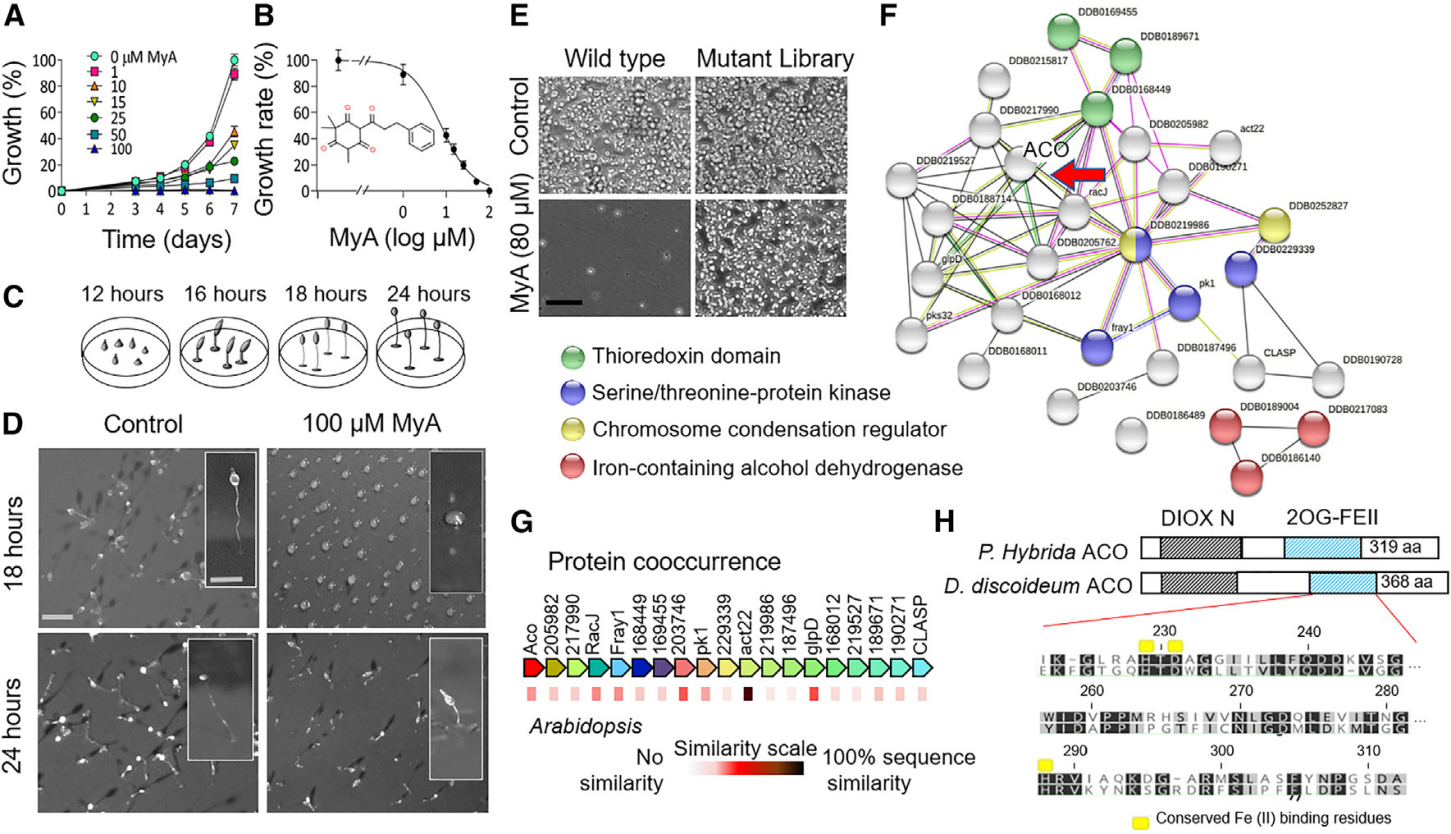
Effect of MyA on *D*. *discoideum* growth and development revealed the ACO protein as a potential target. **(A)** Effect of MyA on cell proliferation of the model system *D*. *discoideum*. Cells were grown under a range of MyA concentrations for 7 days, and MyA produced a concentration-dependent reduction in growth, with no effect at 1 μM, a significant reduction at 10 μM (*P* < 0.05), and complete inhibition at 100 μM. Data are derived from three independent experiments analyzed in triplicate. All samples contained DMSO (0.2%). **(B)** Secondary plot of normalized rate of growth against the log (concentration) of MyA produced an IC_50_ value of 7.1 μM. Insert shows MyA structure. **(C)** Schematic representation of wild-type developmental phenotypes over time under control conditions, showing different stages of development. **(D)** Developmental phenotypes in the absence of MyA (control), showing fruiting body morphology at 18 and 24 h from top-down view and individual fruiting bodies (side view insert). The presence of MyA (100 μM) produced a developmental delay of approximately 6 h that was restored following extended times (Supplemental Figure 1). Images are representative of three independent experiments. Scale bar, 10 mm for the top-down view and 0.5 mm for the single fruiting body. **(E)** A mutant library screen was used to identify potential molecular targets of MyA in *D*. *discoideum*. Both wild-type and mutant-library *D*. *discoideum* cells showed growth in solvent-only conditions. In the presence of MyA (80 μM), after three rounds of screening (72 h per screen), wild-type cells did not proliferate, but a range of mutants showed continued proliferation (resistance). **(F)** STRING analysis of MyA-resistant mutants identified network connectivity relating to MyA function and highlighted ACO within this resistant population. **(G)** Protein co-occurrence was used to identify encoded proteins with plant orthologs, including ACO. **(H)** The *D*. *discoideum* and *Petunia × hybrida* ACO proteins share a common size and domain structure, including the DIOX-N (non-heme dioxygenase in morphine synthesis N-terminal) and 2OGFeII (2OG and Fe(II)-dependent oxygenase containing) domains, and show conserved catalytic residues necessary for Fe (II) binding (*P*. *hybrida* H229, D231, H287), consistent with orthologous functions.

To investigate the molecular mechanism of MyA, we first quantified the effect of MyA on unicellular proliferation (Figure 1A) in the model organism *D*. *discoideum*. In this model, cells divide by binary fission in nutrient-rich media, initially with a lag phase (0–120 h) and then with an exponential phase. MyA treatment caused a concentration-dependent reduction in unicellular proliferation, with significant reduction at 10 μM (*P* < 0.05) and a block in proliferation at 100 μM (Figure 1A) (defined as no significant difference from the starting cell number). Secondary plot analysis revealed an IC_50_ of 7.1 μM (95% confidence interval, 5.7–8.8 μM) (Figure 1B). These data demonstrate that *D*. *discoideum* cell proliferation is sensitive to MyA treatment and validate the use of a library screen to identify mutants resistant to this effect for exploration of MyA bioactivity.

Because *D*. *discoideum* also exhibits multicellular development and this process is often sensitive to bioactive compounds (Chang et al., 2012; Waheed et al., 2014; Perry et al., 2020; Damstra-Oddy et al., 2021), we also investigated the effect of MyA on development. Wild-type *D*. *discoideum* cells aggregate and differentiate over a 24-h period to form a multicellular fruiting body consisting of a spore head, a stalk, and a basal disk (Figures 1C and 1D). In the presence of MyA (100 μM), a concentration at which cell proliferation is blocked, cells aggregated to form tipped mounds in a similar manner to wild-type cells, equivalent to approximately 12 h of development, and later stages of development were delayed (Figure 1C and 1D). Following extended incubation (48 h), the delay in development following MyA exposure was overcome, leading to wild-type fruiting-body morphology (Supplemental Figure 1). These results suggest that high-dose MyA is not lethal but instead triggers a specific delay in development, likely caused by inhibition of a molecular target required for development rather than a generalized toxic effect.

### Identification of ACO as a potential MyA target controlling bioactivity

To identify a potential molecular target of MyA in *D*. *discoideum*, we investigated a library of insertional mutants to identify cells that showed reduced sensitivity to the cell proliferation inhibition caused by MyA treatment. A library of 17 800 mutants with approximately 8000 unique ablated genes was subjected to 3 rounds of MyA exposure (72 h each) at a concentration that inhibited wild-type cell proliferation (80 μM) (Figure 1E). The abundance of each mutant in the library was analyzed using next-generation sequencing of pooled cell populations prior to and after MyA selection (Gruenheit et al., 2021) (Supplemental Figure 2). Mutants that showed a change in abundance specific to MyA treatment were identified by normalizing mutant read counts against those of control groups. This approach identified a total of 112 mutants that were resistant and 8 mutants that were hypersensitive to MyA treatment (Supplemental Figure 3).

To prioritize the potential MyA-resistant mutants for further study, and because the MyA–target interaction is likely to have effects on the interaction partners of the target, we used STRING analysis to highlight common networks in the identified mutants. STRING analysis is a technique for identification of protein networks and protein–protein interactions within a large group of proteins (Figure 1F, Supplemental Figure 4) (Zhao et al., 2019). This analysis identified a single large cluster of 26 proteins that included thioreductase domain–containing proteins, chromosome condenser proteins, and signal transduction proteins (S/T kinases). Further examination of proteins in this cluster using co-occurrence analysis identified proteins with high sequence similarity to *A*. *thaliana* orthologs (Figure 1G). Of particular interest was a mutant that had lost a likely 1-aminocyclopropane-1-carboxylate (ACC) oxidase (ACO) enzyme (Dictybase identifier: DDB_G0277497). This enzyme catalyzes the rate-limiting step of ethylene synthesis in plants (Kende, 1989; Houben and Van de Poel, 2019) and is involved in the release of seed dormancy, promotion of germination, and plant growth (Corbineau et al., 2014).

Analysis of the *D*. *discoideum* ACO protein suggested that it was a likely ortholog of the plant ACO protein from *Petunia* × *hybrida* (PDB: 1WA6, Accession: AAC37381) (Figure 1H). Both proteins are of similar size (368 and 319 aa, respectively), with an overall sequence similarity of 39.7%. They have a common domain structure, with conserved 2-oxoglutarate (2OG) and Fe (II) dependent oxygenase superfamily domains (2OG-Fe(II)) necessary for oxidation of organic substrates such as ACC, as well as conserved Fe(II) binding residues required for enzyme function. Cladistic analysis of ACO proteins confirmed that the identified *D*. *discoideum* ACO protein was closely related to the *Arabidopsis* ACO1 protein (NM_127517.5) (Supplemental Figure 5). These characteristics suggested that the *D*. *discoideum* ACO protein might function in ethylene synthesis in *D*. *discoideum* (Amagai, 1987).

### Molecular modeling analysis suggests direct binding of MyA to ACO

**Figure 2:**
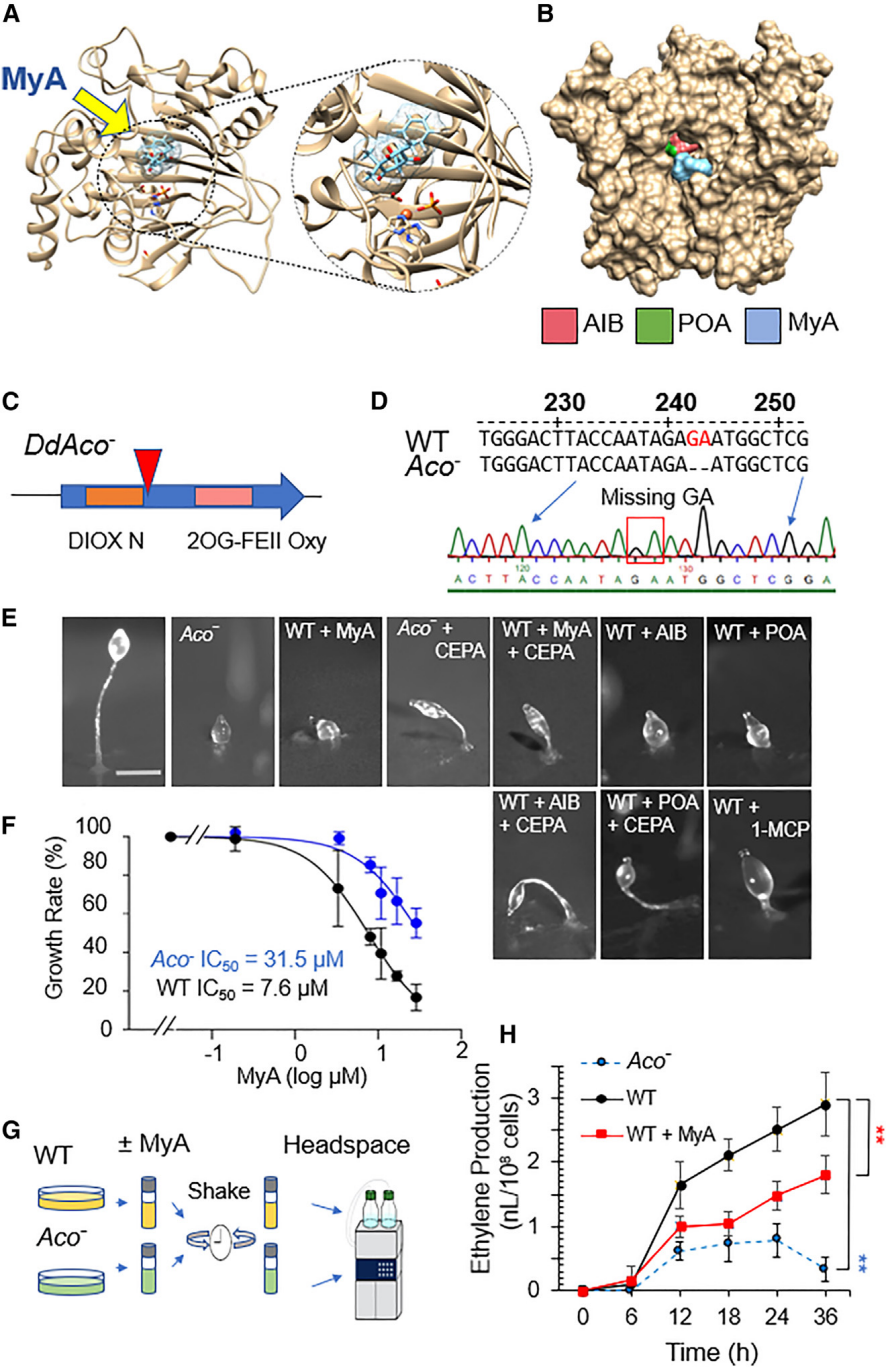
The *D*. *discoideum* ACO ortholog regulates development and growth effects of MyA through loss of ethylene. **(A)** Predicted tertiary structure of *D*. *discoideum* ACO enzyme shown in ribbons and MyA (light blue) binding adjacent to the active site, generated using Phyre2, with a close-up image showing MyA binding in relation to the active site residues (His 229, Asp 231, and His 287) drawn in ball and stick form, with the central Fe(II) shown in orange adjacent to MyA. **(B)** Space-filling model of the *D*. *discoideum* ACO enzyme, shown with binding by existing ACO inhibitors AIB (pink), POA (green), and MyA (light blue) within the catalytic pocket. **(C)** The *Aco*^−^ mutant was created in *D*. *discoideum* by CRISPR to ablate the encoding gene. **(D)** A two base-pair deletion leading to a frame shift. **(E)** Upon starvation, *D*. *discoideum* cells enter a well-defined developmental program in which cells aggregate over 24 h, forming a range of defined structures. Analysis of *D*. *discoideum* development revealed that untreated wild-type cells formed a mature fruiting body at 20 h. By contrast, the *Aco*^–^ mutant was blocked at this time point at the tipped mound stage (equivalent to 12 h of development), similar to the wild type after MyA (100 μM) treatment. In both cases, the developmental block was partially rescued by exogenous ethylene produced by CEPA treatment. A similar developmental delay was produced by two structurally distinct ACO inhibitors, AIB (10 mM), and POA (50 μM), and the effects of both were partially rescued by exogenous ethylene produced by CEPA treatment. A similar developmental delay was observed in the presence of the ethylene receptor inhibitor 1-MCP. Images are representative of three independent experiments. **(F)** Analysis of the sensitivity of the wild type and *Aco*^*−*^ mutants to the effects of MyA on cellular growth indicates partial resistance in the mutant, reflected by an increase in the IC_50_ value of the mutant (shown as mean ± SEM from three independent experiments analyzed in triplicate). **(G)** To measure ethylene production in *D*. *discoideum*, wild-type cells in the absence or presence of MyA (500 μM) or *Aco*^−^-null cells (2 × 10^8^) were starved in collection vials shaken at 140 rpm over 36 h, and ethylene levels in the head space were monitored by GC/MS analysis. **(H)** Ethylene production increased over time and was reduced in the presence of MyA and in the *Aco*^*−*^ mutant. Ethylene production was quantified against a standard curve of known ethylene concentrations. Data are derived from three to five independent experiments analyzed in triplicate and are shown as mean ± SEM. Statistical analysis was performed using ANOVA and Dunnett’s post hoc test for wild type (WT) against WT + MyA (red) or *Aco*^−^ (blue) cells at 18 h post treatment. ∗∗*P* < 0.01.

To investigate a potential direct mechanism of MyA-dependent ACO inhibition, we used a range of molecular modeling techniques (Figure 2A and 2B). The tertiary structure of the *D*. *discoideum* ACO protein was predicted with Phyre2 using the closest available crystal structure as a template (*Petunia × hybrida* ACO: PDB: 5LUN; Uniprot 1WA6) (Figure 2A). MyA was predicted to bind directly to the *D*. *discoideum* ACO protein in the key Fe^2+^ dioxygenase domain, which is required for catalytic activity and in which the substrate ACC binds directly to Fe^2+^ via its carboxylate and amino groups during catalysis, to block substrate access to the facial triad that is essential for ACO activity. Further analysis of this binding using a space-filling model (Figure 2B, Supplemental Figure 6) showed that two structurally distinct ACO inhibitors, 2-aminoisobutyric acid (AIB) and pyrazine-2-carboxylic acid (POA) (Satoh and Esashi, 1980; Sun et al., 2017), bind to the same region as MyA within the substrate binding pocket.

### ACO regulates the effects of MyA on cell proliferation and development

To investigate the cellular and developmental role of the *D*. *discoideum* ACO protein, we initially ablated the corresponding gene using CRISPR technology (Sekine et al., 2018) (Figures 2C, 2D, and Supplemental Figure 7). We analyzed the resulting mutant to reproduce the developmental inhibitory effect of MyA and provide resistance to the cell proliferative inhibition. Because MyA blocks development and cell proliferation, we assessed the effect of ACO loss on both these processes. In these experiments, wild-type cells formed mounds at 12 h and mature fruiting bodies with a round spore head, held aloft by a stalk, and a basal disk at 20 h (Figure 2E). By contrast, the *Aco*^−^ mutant showed delayed (multicellular) development after 12 h at the mound stage by around 6 h. Interestingly, this developmental defect was identical to that observed after treatment of wild-type cells with MyA (100 μM). We repeated the development assays in the presence of exogenous ethylene provided through the breakdown of 2-chloroethylphosphonic acid (CEPA; Ethephon) (Warner and Leopold, 1969) and found that the developmental delays caused by both MyA treatment and ACO loss were partially rescued by addition of ethylene (Figure 2E). A similar developmental delay was also evident after treatment with two structurally distinct ethylene inhibitors, AIB and POA (Satoh and Esashi, 1980; Sun et al., 2017), that were also partially rescued by CEPA. Likewise, the ethylene receptor inhibitor 1-methylcyclopropene (1-MCP) also delayed development (Vanderstraeten et al., 2019). These data suggest that the *D*. *discoideum* protein is a functional ACO enzyme that produces ethylene necessary for timely late development and that the bioactivity of MyA in *D*. *discoideum* development occurs through an ACO-dependent block in ethylene production. We further analyzed the effect of MyA on cell proliferation (Figure 2F). We found a four-fold reduction in potency in the *Aco*^−^ mutant compared with wild-type cells (to a half-maximal inhibitory concentration [IC_50_] of 31.5 μM). This result was consistent with a role for the ACO protein in the mechanism of MyA, as loss of the enzyme reduced the sensitivity of cell proliferation to MyA inhibition.

To provide a quantitative analysis of the developmental effects of MyA and confirm the ethylene-dependent rescue of these effects, we monitored the expression of key developmentally regulated genes (Supplemental Figure 8) in the absence and presence of MyA (Iranfar et al., 2003). In these experiments, wild-type cells were induced to develop on nitrocellulose filters for the indicated time periods under solvent-only conditions, in the presence of MyA (100 μM), or in the presence of MyA and CEPA. Developmental gene expression for early aggregation was assessed by qPCR using (*cs*A), which encodes a key cell–cell adhesion protein in early aggregation and development (Coates and Harwood, 2001), *car*A, which encodes a G-protein coupled cyclic AMP (cAMP) receptor that mediates responses to cAMP (Johnson et al., 1992), and Aco. Expression of *cs*A peaked at around 4 h in untreated wild-type cells; this expression peak was delayed upon MyA treatment and rescued by exogenous ethylene. A similar delay in peak expression time of *car*A after MyA treatment was also rescued by exogenous ethylene. Interestingly, Aco expression increased after 8 h and peaked at 12 h, consistent with a role in development at the mound stage. Thus, delay in the timing of developmental marker expression by MyA treatment was rescued by addition of exogenous ethylene. These experiments confirm a MyA-dependent delay in *D*. *discoideum* development that is rescued by exogenous ethylene application, consistent with a role for MyA in blocking ethylene production dependent on ACO (Supplemental Figure 8).

### MyA treatment reduces ethylene production in *D. discoideum*

To further assess a role for ethylene production in *D*. *discoideum* and the effects of MyA on this process, we used gas chromatography/mass spectrometry (GC/MS) to quantify ethylene production (Figure 2G). In these experiments, wild-type cells in the presence or absence of MyA (500 μM) or *Aco*^−^ cells were starved in phosphate buffer in sealed small flasks with limited head space for 36 h with shaking. Headspace gas was sampled at 6 h intervals and analyzed by GC/MS (Figure 2G). Wild-type cells showed a rapid increase in ethylene production after 6 h (Figure 2H), consistent with peak *acoA* expression levels at 12 h, and levels increased further up to 36 h. MyA treatment reduced ethylene production, causing a 39.5% decrease in ethylene production at 12 h that was maintained up to 36 h (37.9% decrease). Similarly, *Aco*^−^ cells also showed a 62.4% decrease in ethylene production after 12 h that remained low throughout the analysis. These findings show that *D*. *discoideum* cells produce ethylene during starvation and that ethylene production is reduced in the presence of MyA, consistent with MyA-dependent inhibition of ethylene signaling via ACO inhibition.

### Conservation of the MyA binding site in the plant ACO enzyme

**Figure 3:**
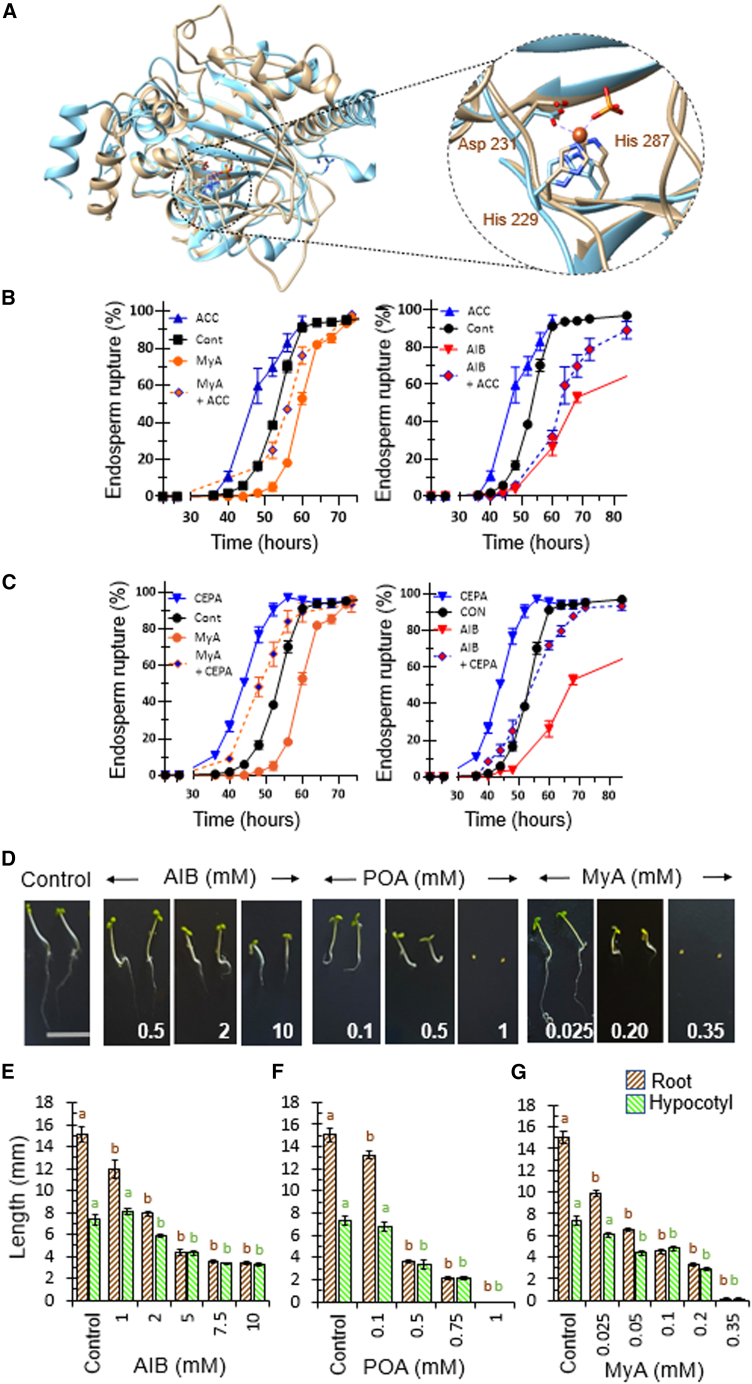
MyA reproduces the effects of established ACO inhibitors on *A*. *thaliana* seed germination and early seedling growth. **(A)** Superimposed structural comparison of *D*. *discoideum* ACO (gold) and *Petunia* ACO (light blue); both proteins feature a double-stranded helix jellyroll fold surrounded by alpha helices. A close-up image shows the conserved catalytic triad adjacent to the predicted MyA binding site. All 3D structures were visualized in Chimera. **(B and C)** To assess the role of MyA in seed germination, after-ripened *A*. *thaliana* Col-0 seeds were incubated in the presence of AIB (up to 100 mM) or MyA (0.5 mM), ACC (1 mM) or CEPA, or combinations at 20°C under continuous white light. Seed germination was measured as the time to endosperm rupture, and AIB produced a dose-dependent reduction in endosperm rupture (Supplemental Figure 9). Both MyA and AIB (20 mM) delayed endosperm rupture, and this effect was partially restored by **(B)** the addition of ACC and **(C)** exogenous ethylene following CEPA treatment. Data are derived from two independent experiments, each with triplicate petri dishes containing 30–50 seeds each, and are shown as mean values ± SEM. **(D)** To assess the role of MyA in seedling growth, *A*. *thaliana* Col-0 seedlings were incubated in a range of AIB (up to 10 mM), POA (up to 1 mM), or MyA (up to 0.35 mM) concentrations under low-light conditions (12-h light/dark cycles), and root and hypocotyl growth were recorded. **(E–G)** Quantification of dose-dependent reductions in root growth (brown) and hypocotyl growth (green) following AIB, POA, and MyA treatment, with high concentrations of POA and MyA blocking seed germination (scale bar, 15 mm). Statistical analysis was performed using ANOVA and Dunnett’s post hoc test for untreated (control) against AIB, POA, and MyA treatments. Different letters indicate statistically significant differences of treatments versus control (*P* < 0.01). Data are derived from three independent experiments, with at least five seedlings per experiment, and are shown as mean ± SEM.

To investigate whether MyA inhibits ACO by a conserved mechanism in plants, we analyzed structural conservation between the *D*. *discoideum* and plant ACO enzymes. The *D*. *discoideum* and *P*. *hybrida* ACO enzyme structures were superimposed (Figure 3A) (Zhang et al., 2004), revealing a common tertiary structure featuring a double-stranded-helix jellyroll fold surrounded by alpha helices, with a root-mean-square deviation of 1.016 Å over 282 aligned Ca atoms. This analysis also identified high 3D structural conservation between the catalytic Fe^2+^ dioxygenase domains and binding amino acids. Thus, MyA was predicted to bind directly within the catalytic pocket of the plant ACO enzyme.

### MyA treatment inhibits ethylene-regulated processes in *A. thaliana* seeds and seedlings

Because seed germination of *A*. *thaliana* and other plant species is promoted by ethylene (Linkies et al., 2009), we initially investigated the role of ethylene production and MyA activity in seed germination by assessing endosperm rupture (Figures 3B and 3C). In these experiments, we used after-ripened (non-dormant) *A*. *thaliana* Col-0 seeds treated with AIB (up to 100 mM) or MyA (0.5 mM), alone or in combination with the ACO substrate ACC (10 μM) or CEPA. AIB treatment produced a dose-dependent delay in endosperm rupture (Supplemental Figure 9), indicating a critical role for ethylene in this process. MyA treatment delayed endosperm rupture, inhibiting germination (Figure 3B), (Oracz et al., 2012; Voegele et al., 2012). This inhibition also occurred with the ethylene inhibitor AIB and was rescued by simultaneous ACC treatment (Figure 3B). Similarly, the delayed endosperm rupture caused by MyA or AIB treatment was also reversed by exogenous ethylene provided by CEPA (Figure 3C). Taken together, these findings support a mechanism in which MyA inhibits ethylene-dependent responses involved in seed germination.

To assess a role for MyA in inhibiting ethylene responses in seedlings, we initially compared the effects of the established ethylene inhibitors AIB and POA (Satoh and Esashi, 1980; Sun et al., 2017) on *A*. *thaliana* root and hypocotyl growth with those of MyA treatment (Figures 3D–3G). In these experiments, *A*. *thaliana* seeds were germinated and grown for 24 h under low-light conditions (3 μmol m^−2^ s^−1^) for 6 days, and root and hypocotyl lengths were recorded (Figure 3D). Consistent with previous reports (Satoh and Esashi, 1980; Merchante and Stepanova, 2017; Vanderstraeten et al., 2019), AIB and POA treatments produced a dose-dependent decrease in root and hypocotyl lengths (Figure 3D–3F). MyA treatment also reduced both root and hypocotyl extension, but with greater potency than the established ethylene inhibitors (Figure 3G). These data confirm a consistent effect of established ethylene inhibitors and MyA on *A*. *thaliana* seedling growth.

Further analysis of MyA (and AIB) effects on *A*. *thaliana* root and hypocotyl growth involved combination treatments with ACC or CEPA. Both ACC and CEPA treatments reduce plant root and hypocotyl growth (Lopez Nicolas et al., 2001; Vanderstraeten et al., 2019), indicating that either a block or an over-supply of ethylene in seedlings inhibits growth. We show that ACC (5 and 10 μM) treatment reduced root and hypocotyl growth (Supplemental Figure 10), and this effect was significantly reversed by MyA (25 μM) and AIB (2 mM) (*P* < 0.001 for both), consistent with the inhibition of ethylene response by both MyA and AIB (Naing et al., 2022). By contrast, because CEPA treatment provided exogenous ethylene independently of ACO activity, high MyA levels (300 μM) reduced root and hypocotyl growth, and this effect was partially reversed by CEPA treatment (Supplemental Figure 10). These data are consistent with inhibition of plant ethylene production by MyA (and AIB) and partial reversal of this inhibition by exogenous ethylene production.

**Figure 4:**
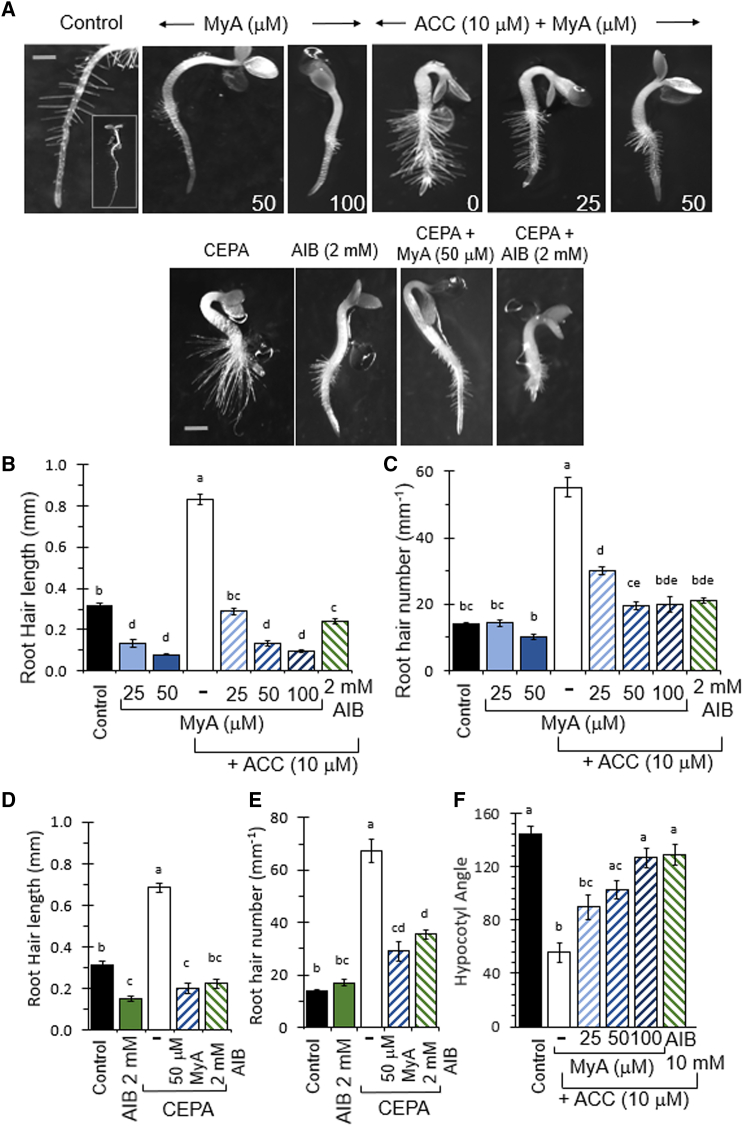
MyA blocks ethylene-dependent processes in plants. **(A)** Root hair formation in *A*. *thaliana* seedlings was assessed in the absence or presence of ACC (10 μM) or CEPA and following combinatory MyA (μM) or AIB (2 mM) treatment using seedlings grown for 3 days. MyA reduced root hair formation, ACC and CEPA treatment increased root hair length and number, and these effects were reversed by MyA or AIB treatment. Scale bar, 0.2 mm. **(B)** MyA treatment reduced root hair length, ACC treatment increased root hair length, MyA produced a dose-dependent inhibition of this effect, and AIB (2 mM) reproduced the effect of MyA on root hair length in combination with ACC treatment. **(C)** ACC treatment increased root hair number, MyA inhibited this effect, and AIB (2 mM) reproduced the effect of MyA on root hair number in combination with ACC treatment. **(D and E)** CEPA increased root hair length and number, and this effect was reduced by MyA (50 μM) and AIB (2 mM). **(F)** Apical hook curvature, part of the ethylene triple response, was assessed after 3 days of seedling growth in 24-h low-light conditions. MyA treatment caused a concentration-dependent decrease in ACC-induced apical hook curvature. Data are derived from three independent experiments analyzed in triplicate and are shown as mean ± SEM, each with five seedlings. Statistical analysis was performed using ANOVA and Tukey’s honestly significant difference test. Different letters denote statistically significant differences (*P* < 0.01).

Because root hair length and number have been shown to increase after ethylene exposure (Feng et al., 2017), we investigated this process using ethylene levels enhanced by ACC or CEPA treatment (Figure 4). In these experiments, MyA reduced root hair length and number in the absence of ACC (Figure 4A-4C). Following ACC treatment (10 uM), root hair length and number increased, and both effects were inhibited after MyA and AIB treatment (Figure 4A-4C). Similar increases in root hair length and number occurred after CEPA treatment (Figure 4D and 4E), and both effects were again reduced by MyA or AIB treatment.

We next analyzed the effect of MyA on hypocotyl hook formation as part of the ethylene triple response, in which treatment of seedlings with ACC (10 μM) results in exaggerated apical hooks (Merchante and Stepanova, 2017) (Figures 4A, 4F, and Supplemental Figure 11). This ACC-induced effect was reversed in a dose-dependent manner after MyA and AIB (2 mM) treatment (Figure 4F). These data provide evidence for a block in ethylene-induced plant responses by MyA, consistent with that observed after AIB treatment.

### Novel structure searches identify potential second-generation ACO inhibitors

**Figure 5:**
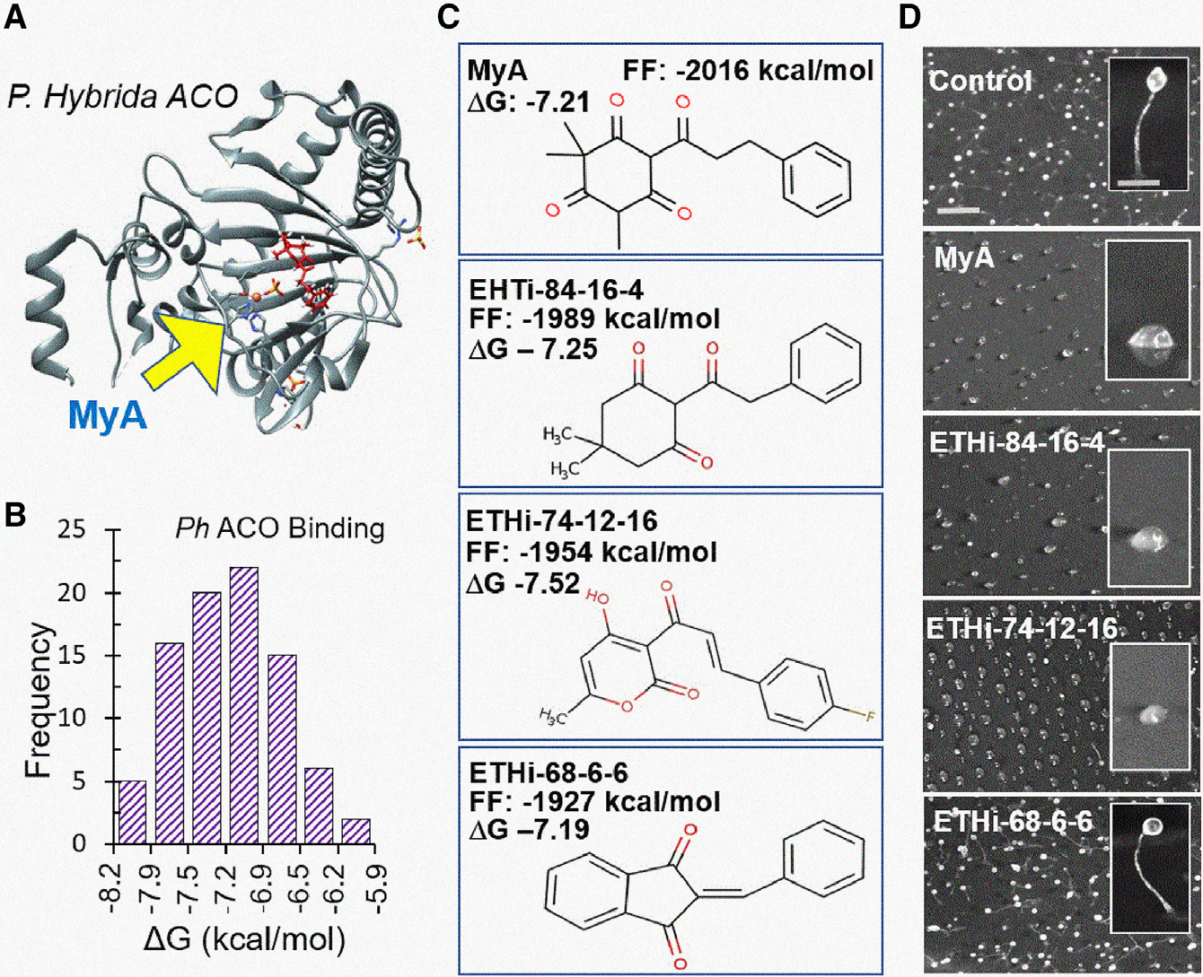
Novel structure searches identify potential second-generation ethylene response inhibitors. **(A)** To mimic the inhibition of ACO associated with binding of MyA within the catalytic pocket adjacent to the iron group, we used an *in silico* modeling approach with the plant (*P*. *hybrida*) ACO enzyme. **(B)** Analysis of a range of chemical structures that contained potential chemophores found in MyA identified compounds that were predicted to bind to the same region of the ACO enzyme as MyA, with a range of binding affinities defined by ΔG values. **(C)** Several structures were identified with estimated full fitness (FF) and ΔG values suggesting potentially enhanced or reduced binding compared with that of MyA (Supplemental Figures S12–S14). **(D)** Bioactivity assays for *D*. *discoideum* development revealed compounds that reproduced the effect of MyA in causing an ethylene-related block in development, although some compounds lacked activity (ETHi-68-6-6). Images are representative of three independent experiments.

Because *in silico* modeling predicted binding of MyA, POA, and AIB to the active site of plant ACO enzymes, we searched for novel structures that also provided this predicted binding (Figure 5). We analyzed 84 compounds containing structural groups found in MyA for binding to the active site pocket of the *P*. *hybrida* (PDB: 1WA6) ACO enzyme (Figure 5A). Strength of binding was estimated by comparing ΔG and full fitness values of each compound with those of MyA. Using this approach, we identified 73 compounds that were predicted to bind to the active site of the plant enzyme, revealing a range of compounds with potentially enhanced binding compared with that of MyA, POA and AIB (Figure 5B and Supplemental Figure 12). To validate these compounds for cellular activity in ethylene inhibition, and because ACO inhibition blocks *D*. *discoideum* development at the mound stage, we analyzed 16 compounds using this bioassay (100 μM) (Supplemental Figure 13) and identified several compounds with strong or partial efficacy compared with that of MyA in this model. A second round of screening using these active compounds and related structures, identified 16 compounds with likely ethylene inhibitory activity (termed ETHi) in this assay (Figures 5C, 5D, and Supplemental Figure 14), although some related compounds lacked this bioactivity (ETHi-68-6-6).

**Figure 6:**
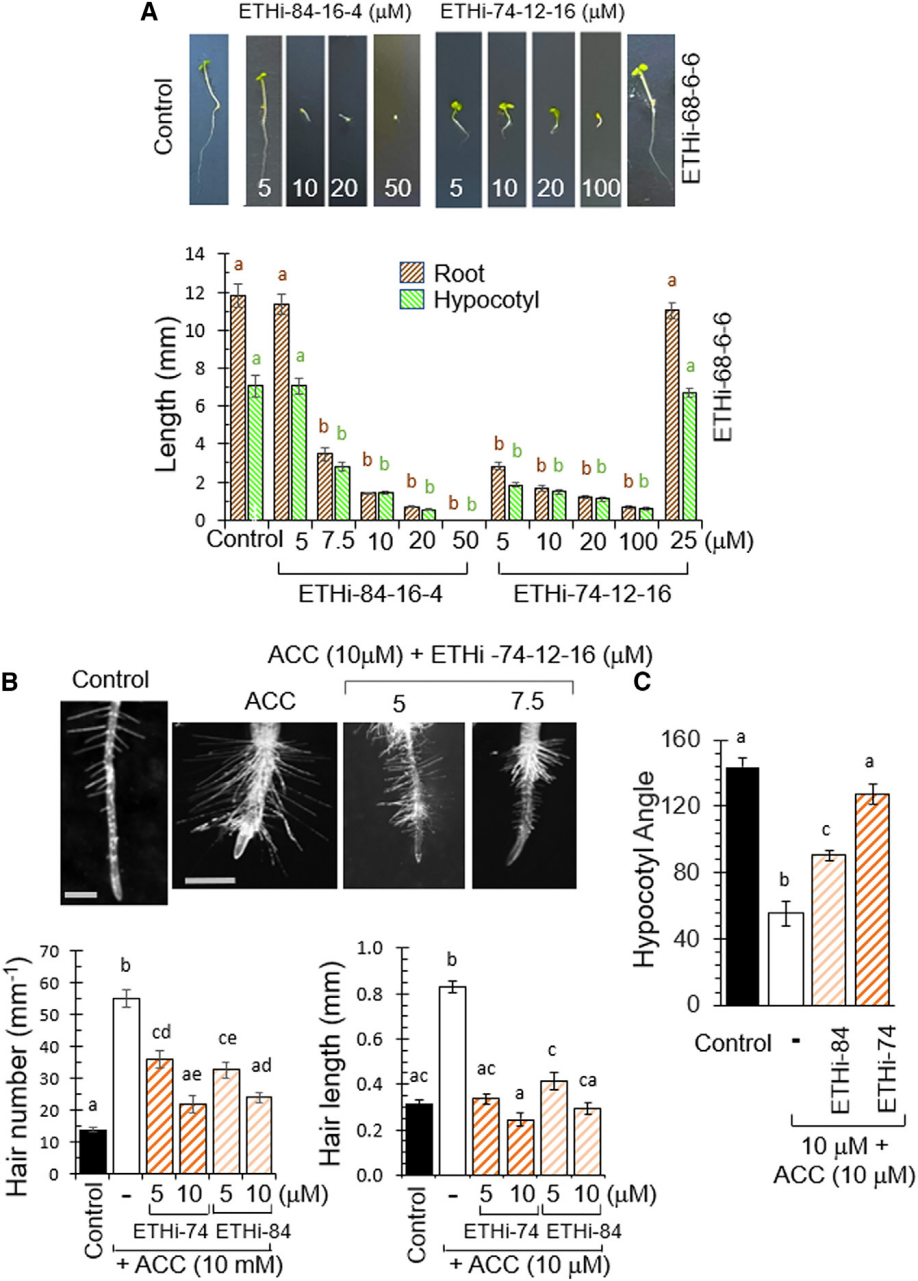
Second-generation ethylene inhibitors based upon MyA show potent ethylene response inhibition. **(A)***A*. *thaliana* Col-0 seedlings were incubated under low-light conditions. Root growth (brown) and hypocotyl growth (green) were recorded at a range of concentrations of compounds predicted to inhibit ethylene response (ETHi-74-12-16 and ETHi-84-16-4) and of a related compound predicted to lack ethylene response inhibition (ETHi-68-6-6) (scale bar, 15 mm). **(B)** Novel compounds were assessed for inhibition of ethylene-dependent increases in root hair number and length. Images are shown for ETHi-74-12-16 only, and quantification is shown for ETHi-74-12-16 and ETHi-84-16-4, demonstrating dose-dependent inhibition of this effect. **(C)** Apical hook curvature, part of the ethylene triple response, was assessed after 3 days of seedling growth in 24-h light conditions. Treatment with ETHi-84-16-4 (ETHi-84) and ETHi-74-12-16 (ETHi-74) decreased ACC-induced apical hook curvature. All data are derived from three independent experiments, each with 10–15 **(A)** or 5 **(B and C)** seedlings, and are shown as average ± SEM. For **(A)**, statistical analysis was performed using ANOVA and Dunnett’s post hoc test for wild type (WT) against WT + treatment. Different letters indicate a statistically significant difference in comparison to control treatments (*P* < 0.01). For **(B and C)**, statistical analysis was performed using ANOVA and Tukey’s HSD. Different letters denote statistically significant differences (*P* < 0.01).

To confirm the efficacy of these potential ethylene inhibitors *in planta*, we analyzed the dose–response effects of selected compounds on root/hypocotyl growth (Figure 6). In this analysis, the compounds showed highly potent inhibition of hypocotyl or root extension (Figure 6A), in one case more than 5340-fold or 1180-fold, respectively (Supplemental Figure 15). Compounds that did not show activity in the bioassay did not reduce root/hypocotyl extension in these assays (Figure 6A), and potent compounds also blocked the ethylene-dependent increase in root hair number and length (Figure 6B). We analyzed the effect of novel compounds on hypocotyl hook formation as part of the ethylene triple response, and the ACC-induced response was significantly reversed following treatment (Figure 6C) (*P* < 0.01). These data confirm the identification of a range of new compounds that potently reproduce the effect of structurally independent ethylene inhibitors in plants and block ethylene-dependent responses.

**Figure 7:**
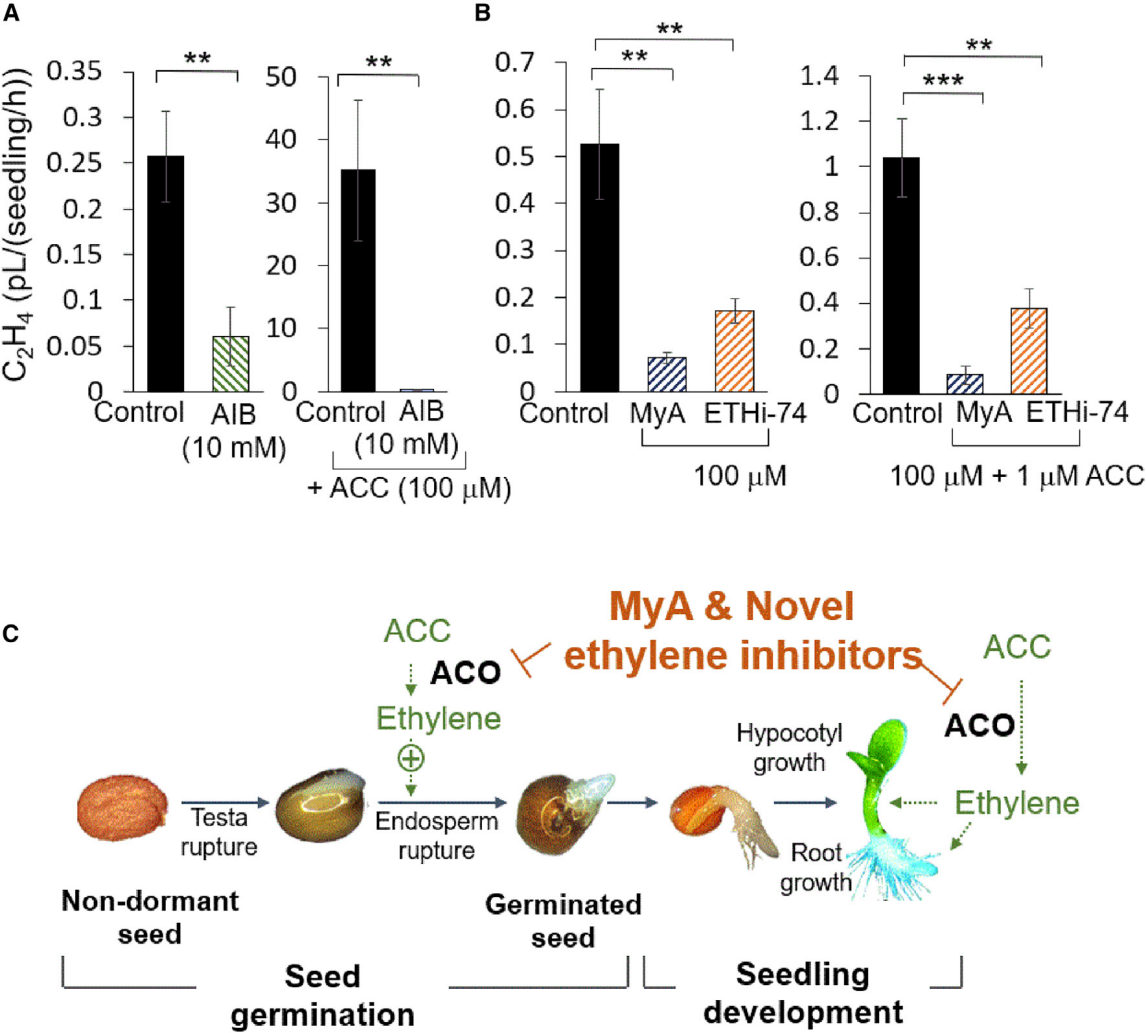
MyA and second-generation ethylene inhibitors reduce ethylene production in *Arabidopsis*. **(A and B)***Arabidopsis* Col-0 seedlings were grown for one day in plates in the light, then transferred to bottles for 2 days in darkness with the indicated concentrations of ACC, AIB, MyA, and ETHi-74-12-16 (ETHi-74). Headspace ethylene was quantified using an ETD300 ethylene detector. **(A)** Under control conditions, seedlings produced ethylene, and treatment with AIB reduced ethylene production. The increased ethylene production following ACC treatment was also reduced by AIB. **(B)** Treatments with MyA and ETHi-74 reduced ethylene production both under control conditions and following stimulation with ACC. Statistical analysis was performed using Student’s *t* test for the untreated control versus MyA, AIB, or ETHi-74 treatment (∗∗*P* < 0.01, ∗∗∗*P* < 0.001). **(C)** Schematic diagram of seed germination and seedling development, indicating a role of MyA and novel ethylene inhibitors in endosperm rupture and root and hypocotyl growth through inhibition of ethylene production.

Finally, we investigated the ability of the potential ethylene inhibitor compounds to inhibit ethylene production *in planta*. In these experiments, *A*. *thaliana* seedlings were grown in 10 mL sealed vials for 2 days, and headspace ethylene was measured. Ethylene production in these assays was reduced by AIB (10 mM) in both the absence and presence of ACC (100 μM) (Figure 7A). MyA or a novel ethylene inhibitor also significantly reduced ethylene production in these assays (Figure 7B) (*P* < 0.001 and *P* < 0.01, respectively). Together, these analyses reveal a mechanism for MyA and novel ethylene inhibitors in blocking ethylene production in seed germination, root and hypocotyl growth, and root hair production, reproducing the effects of established ethylene biosynthesis inhibitors (Figure 7C).

## Discussion

Investigating the mechanisms of action of plant-derived allelochemicals that show desirable phytotoxic effects has been a priority in agriculture and horticulture, as such compounds could be used as bioherbicides or plant growth regulators and to increase crop quality, yield, or resistance to environmental stress (Chen et al., 2020). Identifying allelochemicals that could fulfil these roles is relatively simple due to the easy classification of broad phenotypic effects; however, identifying the direct molecular mechanisms by which these compounds act is more challenging, as plant phytotoxins often have multiple targets (Dayan and Duke, 2020). Identifying such mechanisms enables the subsequent development of new compounds with increased specificity, potency, or safety characteristics. These compounds are often linked with the regulation of plant hormones, as hormones are essential for regulating plant growth and development, and thus plant experimental models are often used in allelochemical research (Dayan and Duke, 2014).

Here, we investigated MyA, an allelochemical that demonstrates phytotoxic activity on seed germination and early seedling growth (Popovici et al., 2011; Oracz et al., 2012), using the model system *D*. *discoideum*. From an unbiased genome-wide chemical genetic screen, we identified the ACO enzyme, which catalyzes a key step in the ethylene biosynthetic pathway, as a potential target for MyA. We showed that MyA reduces *D*. *discoideum* cell proliferation dependent upon ACO activity and delays development, an effect that was reproduced by genetic ablation of the ACO enzyme. Supporting the mechanism of MyA-dependent ACO inhibition, the developmental delay caused by MyA was partially rescued by exogenous ethylene, and a similar delay was produced by the established ACO inhibitors AIB and POA (Amrhein and Wenker, 1979; Sahoh and Esashi, 1983). Finally, both wild-type cells treated with MyA and ACO^−^ mutants showed a decrease in ethylene production in this model.

Interestingly, although several ethylene receptors and signal transduction components of seed plants are conserved and evolved in cyanobacteria and green algae before the ability to synthesize ethylene, far less is known about the evolutionary origin of ACO and thereby ethylene biosynthesis via ACC (Ju and Chang, 2015; Li et al., 2022). The ACO pathway is present in all seed plants, some green algae, some fungi, and the social ameba *Dictyostelium*. In both seed plants and *D*. *discoideum*, ethylene is synthesized from methionine via *S*-adenosyl-l-methionine and 1-aminocyclopropane-1-carboxylic acid (ACC) (Adams and Yang, 1979; Amagai and Maeda, 1992), with ACO acting just after the rate-limiting step in the biosynthetic pathway (Choudhury et al., 2013). Phylogenetic analysis combined with enzyme activity assays suggested that seed plant ACOs may have originated from micro-organisms (Li et al., 2022). This comparison also demonstrated that an active site containing a Fe(II) ligated by three residues (His, Asp, His) is the conserved hallmark of functional ACOs. Adjacent to this conserved catalytic triad is where binding of established ACO inhibitors and MyA occurred in *D*. *discoideum* ACO (Dictybase identifier: GO277497) and *P*. *hybrida* PhACO1 (PDB: 1WA6) (Figure 3). In addition to the identified ACO ortholog (Dictybase identifier: GO277497), there are at least three other potential ACO enzymes in *Dictyostelium* (GO283291, GO286203, and GO286201) that share the 2OG and Fe(II)-dependent oxygenase superfamily protein structure and remain uncharacterized. Furthermore, ethylene plays a key role in inducing sexual development and zygote formation (Amagai, 1987; 2011), and the ethylene receptor inhibitor 1-MCP blocks this process, suggesting that ethylene is essential for both multicellular and sexual development. There are at least nine histidine kinases in *D*. *discoideum*, such as DhKK (GO277887), that share conserved domains and tertiary structures with *A*. *thaliana* ETR1 and may function as ethylene receptors, supporting the existence of an ethylene-dependent signaling pathway (Amagai et al., 2007). In seed plants, ethylene is a potent regulator of many physiological and developmental processes, including seed germination, plant growth, flower senescence, and fruit ripening (Iqbal et al., 2017). Changes in ethylene levels and perception function both directly and in combination with other plant hormones to play a key role in these processes (Santner et al., 2009).

MyA was previously found to interfere with endosperm rupture during the early stages of seed germination by inhibiting endosperm cap weakening (Oracz et al., 2012; Voegele et al., 2012). Regulation of seed germination in response to environmental signals is reliant on a hormonal balance between abscisic acid and gibberellins (Finkelstein et al., 2008) but also on ethylene and auxins (Ghassemian et al., 2000; Linkies et al., 2009). We showed that MyA-induced effects are likely due to inhibition of ACO to reduce ethylene biosynthesis, as previous studies have shown that increased ethylene production during germination is due to increased ACO activity (Ghassemian et al., 2000; Linkies et al., 2009). Application of external ethylene also stimulates germination for a range of plant species, particularly under biotic and abiotic stresses (Corbineau et al., 2014), and germination ability is correlated with ethylene production (Arc et al., 2013). Biomechanical measurements of endosperm weakening during seed germination (Steinbrecher and Leubner-Metzger, 2017), which is promoted by ethylene, are consistent with an effect of MyA in reducing ethylene production. These roles suggest that MyA disrupts the balance between embryo growth potential and covering-layer resistance, thus explaining the mechanisms of MyA-induced germination inhibition through inhibition of ethylene production.

The inhibition of ACO by MyA in *A*. *thaliana* seedlings shown here confirms earlier studies that reported these effects in eudicot and monocot seedlings (Popovici et al., 2010, 2011). We showed that the inhibitory effect of MyA on root and hypocotyl growth was reproduced by exposure to established ethylene biosynthesis inhibitors (AIB and POA) and that both MyA and AIB partially reversed the effects of ACC on root and hypocotyl growth (Vanderstraeten et al., 2019), consistent with a mechanism in which MyA blocks ethylene synthesis and reduces total ethylene production of *A*. *thaliana* seedlings (Figures 7A and 7B). The inhibition of root growth by MyA likely occurs through reducing ethylene production and inhibiting cell proliferation in the root meristem (Qin et al., 2022) or through restricting epidermal cell expansion in the root apical meristem and reducing cell elongation (Ruzicka et al., 2007; Qin et al., 2019). However, it is important to note that the effect of ethylene varies between light and dark conditions; ethylene (and ACC) reduces both root and hypocotyl elongation (Ruzicka et al., 2007) in the dark and in low-light conditions in addition to sucrose supplementation inhibiting hypocotyl growth, but it increases hypocotyl and root extension in high-light conditions (Smalle et al., 1997; Yu and Huang, 2017). Finally, ethylene exposure has been demonstrated to enhance root hair production (Feng et al., 2017), an effect shown here to be blocked by MyA and AIB exposure. Although direct binding of MyA to plant ACO was not demonstrated in this study, a recent paper indicates that MyA treatment does not reduce Aco gene expression (Nakabayashi et al., 2022), ruling out a transcriptional regulatory mechanism and supporting a biochemical mechanism for inhibition of ethylene production by MyA. Thus, these studies indicate that MyA treatment reproduces the effects of established ACO inhibitors and blocks the effects of enhancing ethylene production in plants, confirming a role for MyA in ACO inhibition.

After molecular docking analysis suggested the direct binding of MyA to both the *D*. *discoideum* and seed plant ACO enzymes, we used this method to identify and characterize new structures that might act as ACO inhibitors with enhanced potency. Consistent with other members of the 2OG-dependent dioxygenase superfamily, ACO is a nonheme Fe(II)-containing enzyme (Kawai et al., 2014) that contains a characteristic double-stranded beta helix with a 2-His-1-carboxylate motif consisting of two histidines and an acidic residue (Glu/Asp) required for iron binding. MyA is predicted to bind within the ACC oxidase catalytic domain, suggesting competitive inhibition of ACO with respect to the ACC substrate. This modeling approach was supported by showing direct binding of AIB and POA to the active site of the enzyme, as previously reported for POA (Sun et al., 2017), to explain the molecular basis for this inhibition. Through analysis of binding coefficients for compounds that shared similar functional structures, together with bioassays of *D*. *discoideum* development, we identified a family of compounds that exhibit potent ACO inhibition activity. These compounds share a common structure based around a two-substituted ketone cyclohexene-1,3-dione core that binds within the catalytic cleft of both plant and *D*. *discoideum* ACO enzymes. These compounds also show a large increase in potency relative to AIB and POA in *A*. *thaliana*. Interestingly, their structures show some similarity to existing *p*-hydroxyphenylpyruvate dioxygenase inhibitors, including grandiflorone and the commercial herbicide Sulcotrione (Dayan et al., 2015), that lead to seedling bleaching; however, neither MyA (Oracz et al., 2012; Nakabayashi et al., 2022) nor the new compounds described here cause bleaching (Figures 3 and 6), ruling out this molecular mechanism.

Despite the strong evidence for MyA as an ACO inhibitor, further lines of evidence may indicate other mechanisms. For example, analysis of the *A*. *thaliana* constitutive ethylene-activated mutant *ctr1-1* that mimics high ethylene exposure, showing thickening of hypocotyls and reduced root and hypocotyl growth (Kieber et al., 1993) may not have the seed germination defect following treatment. Furthermore, the relatively high concentration of established ACO inhibitors (AIB, POA) (Sun et al., 2017) may lead to a non-specific outcome. Therefore, the current study does not exclude potential secondary effects of MyA on regulation of other plant functions.

This new family of ACO inhibitors has the potential to function in a range of areas. Their potential uses include reducing senescence in plant cuttings and flowers (Kosugi et al., 1997; Singh et al., 2019), modifying seed germination and weed growth (Hansen and Grossmann, 2000), regulating responses to heat and drought stress that are likely to arise from global warming (Arraes et al., 2015; Li et al., 2020), and reducing susceptibility to infection (Khan et al., 2017). In addition, this mechanism is important in agricultural crops such as rice, in which maintaining an appropriate degree of seed dormancy by inhibiting ethylene production may reduce pre-harvest sprouting and reducing ethylene production may increase starch synthesis and rice grain quality (Tamaki et al., 2015; Shu et al., 2016). In addition, ethylene biosynthesis inhibitors have been proposed as a mechanism for extending the lifespans of vegetables, fruits, and cut flowers (Wills, 2021).

## Methods

### *D. discoideum* cell proliferation-resistance assay

*D*. *discoideum* wild-type cells (Ax3) were grown in still culture conditions in HL5 medium (Formedium, HLB0103) at 22°C under constant light. For growth assays, cells were harvested, and 1 × 10^4^ cells in aliquots of 500 μL were transferred to 24-well plates containing MyA at defined concentrations. Cell growth was assessed after 72 h and then every 24 h. Dose–response curves were created and IC_50_ values determined using non-linear regression analysis. Compound concentrations were empirically determined to identify maximal and minimal effective doses for cell proliferation inhibition.

### *D. discoideum* development assay

Wild-type (Ax3) *D*. *discoideum* cells in the exponential phase of growth were harvested and washed in KK2 buffer (16.2 mM KH_2_PO_4_, 4 mM K_2_HPO_4_). Absorbent pads were placed in 2 mL culture dishes and soaked with 0.5 mL KK2 buffer containing MyA (100 μM) or DMSO. Nitrocellulose filter papers (Millipore, HABP04700) were covered in 1 × 10^7^ cells, placed on absorbent pads (Millipore, AP1004700), and maintained in a humid, dark environment at 22°C for 12–36 h. Fruiting body morphology was recorded at 20 h or indicated time points. For ethylene rescue assays, ethylene was added using CEPA (Sigma, C0143) (Warner and Leopold, 1969), with a final concentration range between 50 μM and 1 mM, in airtight 5 mL petri dishes. Compound concentrations were chosen based upon the maximal effective dose for cell proliferation assays.

### REMI-seq mutant library screen

To identify MyA-resistant mutants, we performed a REMI-seq mutant library screen using 17 823 mutants containing approximately 8000 unique gene knockouts (Gruenheit et al., 2021). In the screen, 5 × 10^5^ *D*. *discoideum* cells from the library were grown in still culture dishes in the presence of MyA (80 μM) for 72 h at 22°C. Cells were harvested, and 5 × 10^4^ cells were re-screened for a total of three rounds of screening. MyA resistance was checked using 5 × 10^5^ wild-type cells expressing GFP combined with 5 × 10^5^ cells from the final round of screening; cells were grown for 48 h in 100 μM MyA. The ratio of wild-type GFP-expressing cells to MyA-resistant cells was calculated using a BD FACSCanto cell analyzer, and MyA resistance was achieved if the percentage of wild-type GFP-expressing cells was below 20% (Supplemental Figure S2).

### Preparation of genomic DNA for Illumina sequencing

To identify the genes ablated in the MyA-resistant cell pools, genomic DNA was collected from three independent screens, using 5 × 10^8^ cells per pool, washed in cold KK2 buffer, and suspended in 30 mL nuclei buffer (40 mM Tris [pH 7.8], 1.5% sucrose, 0.1 mM EDTA, 6 mM MgCl_2_, 40 mM KCl, 0.4% NP-40 substitute, 5 mM DTT). Nuclei were pelleted by centrifugation at 4000×*g* for 30 min at 4°C and suspended in EDTA to a final volume of 150 μL and an EDTA concentration of 100 mM (pH 8.0); sodium lauryl sarcosyl (100 μL of 10%) was added, and the mixture was incubated at 55°C for 20 min before addition of ammonium acetate (375 μL 4 M) prior to centrifugation (20 000×*g*, 15 min, 4°C). DNA in 450 μL of supernatant was transferred to absolute ethanol (900 μL) and mixed. The samples were centrifuged (20 000×*g*, 10 min, 4°C), and the remaining pellets were washed with 70% ethanol before suspension in elution buffer (49 μL) and RNAse (1 μL, Invitrogen, AM2286).

To prepare DNA fragments for Illumina sequencing, 1 μg genomic DNA was used for each sample and excised using MmeI and I-SceI (New England Biolabs). Following digestion, indexed adapters were ligated to the digested DNA, with different combinations of D7 and D5 indices used to tag each sample. Ligation reactions were performed overnight at 16°C using total digested DNA, 2 ng/200 ng of D7 and D5 pre-annealed indexed adapters, and 400 units of T4 DNA ligase. DNA of interest was amplified using PCR with primers specific to the D7 and D5 adapter regions, and pooled samples were then separated by agarose gel electrophoresis. The band of interest (183 bp) was excised, the DNA was extracted (QIAquick, Qiagen), and the resulting samples were analyzed using a Qubit 3.0 Fluorimeter and sequenced on an Illumina MiSeq system with a MiSeq Reagent Kit v3 (75 cycles) or the NextSeq 500 Sequencer with a High Output Kit v2 (75 cycles).

### CRISPR acoA gene ablation

Gene knockouts of *ACO* were created as described previously (Sekine et al., 2018). CRISPR guide sequences were designed and checked for potential off-target sites using the CRISPOR online tool (http://crispor.tefor.net). Three unique oligo pairs were designed and cloned into a pTM1285 vector, followed by electroporation into wild-type AX2 *D*. *discoideum* cells. Transformed cells were incubated with G418 at a final concentration of 10 μg/mL for 3 days before serial dilution onto SM plates to create clonal isolates. Each clonal isolate was screened by PCR amplification of the *ACO* gene region and sequenced to confirm frame-shift mutations.

The CRISPOR online tool was used to create CRISPR-Cas9 guide sequences specific to the Dd.*ACO* gene. Three guide pairs were designed as follows:

Oligo 1A: 5′ AGCATGGGACTTACCAATAGAGAA 3′

Oligo 2A: 3′ TTCTCTATTGGTAAGTCCCACAAA 5′

Oligo 1B: 5′ AGCATCGGTAAGAGATAACCATTC 3′

Oligo 2B: 3′ AGCCATTCTCTATTGGTAAGCAAA 5′

Oligo 1C: 5′ AGCAAATAAAGCATGTAAAGAGTA 3′

Oligo 2C: 3′ ACTTTGAAGAATCCGAGCCACAAA 5′

Each target-specific oligo pair was annealed and phosphorylated by incubating 1 μL Oligo 1/2 (100 μM) with 0.5 μL T4 polynucleotide kinase and 1 μL 10 × T4 DNA ligase buffer (Thermo Fisher Scientific). The reaction mixture was incubated at 37°C for 30 min, 95°C for 5 min, and then ramped down to 25°C at a rate of 1°C every 10 s using a peqSTAR thermocycler. We used 2 μL of 1/250 diluted annealed guide sequences, which were digested and ligated with 100 ng CRISPR-Cas9 vector (pTM12853), in the following 20 μL reaction mixture and conditions: 2 μL 10× FASTDigest buffer (Thermo Fisher Scientific), 1 μL 1,4-dithiothreitol (Sigma-Aldrich, final concentration of 1 mM), 1 μL FastDigest Bpil (Thermo Fisher Scientific), and 1 μL T4 DNA ligase (5 units/μL, Thermo Fisher Scientific) for six cycles of 37°C for 5 min and 23°C for 5 min.

Following digestion and ligation of each individual oligo pair into pTM1285 plasmids, the reaction mixture was transformed into chemically competent *Escherichia coli* cells as described previously. Successfully transformed plasmids were purified using a miniprep kit and transfected into *D*. *discoideum* cells as described previously. After incubation in G418 for 3 days, isogenic strains were isolated by spreading serial dilutions of cells onto SM agar plates supplemented with topological *Raoultella planticola*. To confirm sequence mutations, target gene sequences were amplified by PCR using gene-specific primers. Amplified PCR products were purified using a QIAquick PCR purification kit (Qiagen) following the manufacturer’s protocol and sequenced using Eurofins TUBESEQ service.

### qPCR

For analysis of *D*. *discoideum* developmental gene expression, we used 2 × 10^7^ cells incubated on nitrocellulose filters soaked in KK2 in the presence of MyA or DMSO; cells were collected at the indicated time points in KK2. RNA was isolated as described previously (Ludtmann et al., 2014) (Qiagen, 74101), and contaminating DNA was removed (DNA-free DNA Removal Kit, Thermo Fisher Scientific, AM1906). cDNA was generated from 2 μg RNA (RevertAid First Strand cDNA Synthesis Kit, Thermo Fisher Scientific, K1622) and random hexanucleotide primers. Quantitative PCR reactions were performed in triplicate with three independent biological samples (cells derived from independent cell populations) using a Qiagen Rotor-Gene Q platform and SYBR master mix (Thermo Fisher Scientific, 4364344). Each independent experiment consisted of RNA extracted from cells harvested and pooled from three filter papers. Cycling conditions were as follows: 95°C for 10 min and 30–40 cycles of 95°C for 15 s, 45°C for 30 s, and 62°C for 1 min. Quantification was performed using an absolute quantification method with reference gene plasmids generated using a TOPO TA cloning kit (Thermo Fisher Scientific K450002) and a standard curve generated from known serial dilutions. Primer sequences for acoA (Dictybase ID: DDB_G0277497), carA (Dictybase ID: DDB_G0273397), and csaA (Dictybase ID: DDB_G0289073) were as follows (sequences are shown 5′ to 3′):

Qpcr_acoA_F: Qpcr Aco AGAGAATGGCTCGGATTCTTC.

Qpcr_acoA_R: Qpcr Aco TTCCTCTTGTTCACGGTTG.

Qpcr_carA_F: Qpcr carA ATGATGATAAAGAAGATGAAGATGAACC.

Qpcr_carA_R: Qpcr carA CCAGCACTCAATATTCTCC.

qpcr_csaA_F: Qpcr csA TCGTGCCAAATACAATCGCTGGTG.

qpcr_csaA_R: Qpcr csA TGGGCTTGAGGTTCCCCATGGT.

### Analysis of protein sequences

Full *D*.*discoideum* protein sequences were identified using the Dictybase online resource, with potential *A. thaliana* identified using BLAST analysis. Functional domains were identified using annotations from InterPro (https://www.ebi.ac.uk/interpro/), and STRING analysis (STRING v11: https://string-db.org/) was used to identify protein–protein interactions and protein co-occurrences.

### Analysis of ethylene production in *Dictyostelium*

Ethylene production was measured from 2 × 10^8^ *D*. *discoideum* cells placed into a 2 mL GC/MS collection vial and shaken at 140 rpm for 0–36 h. Internal ethylene concentrations (parts per million) were generated from a standard curve using known concentrations of ethylene (Sigma Aldrich, UK). Each biological replicate was an independent GC/MS collection vial measured in triplicate. GC/MS analysis was performed using an Agilent 7820A GC (Agilent) equipped with on-column injection and connected to an Agilent 5977B (Agilent) quadrupole mass spectrometer. Headspace gas was manually injected with a 10 μL gas-tight syringe (Trajan SRG) into the GC inlet in splitless mode using a Merlin Microseal septum (Merlin Instrument Company). The GC was equipped with a GS Carbon Plot column (30 m × 320 μm with a carbon-layer stationary phase; Agilent J&W). To remove nitrogen contamination, a selected ion monitoring method set to select ions at 26 m/z and 27 m/z was used with a dwell of 15 ms and an electron ionization potential of 70 eV.

### *In silico* modeling of ACO and MyA binding

For 3D structural analysis and modeling of the *D*. *discoideum* ACO protein, Phyre2 (http://www.sbg.bio.ic.ac.uk/phyre2/) was used with the closest available crystal structure (PDB: 5LUN) as a template. Potential binding sites of MyA and related known ACO inhibitors were analyzed by automated molecular docking performed with the web-based SwissDock program (www.swissdock.ch/docking) using the generated Phyre2 3D structure and the “accurate” parameter; other parameters were set to default values. Modeling and docking results were visualized using UCSF Chimera v1.12 software.

### Plant material, seedling growth conditions, and germination assays

To analyze seed germination, *A*. *thaliana* accession Col-0 was grown in a glasshouse with a temperature maintained close to 25°C/20°C and 16 h of light provided daily. For germination assays, 30–50 after-ripened seeds were plated in a Petri dish (6-cm diameter) onto a filter paper (MN713, Macherey-Nagel) moistened with 0.8 mL of autoclaved deionized water or aqueous solutions of compounds at the concentrations indicated. At least three replicate plates were incubated in a growth chamber (MLR-352, Panasonic) set at 20°C with continuous light (approximately 100 μmol m^−2^ s^−1^). Testa and endosperm rupture was scored over time using a binocular microscope as described previously (Linkies et al., 2009). MyA was dissolved in DMSO, CEPA in methanol, and ACC (Sigma Aldrich), AIB (Sigma Aldrich), and POA (pyrazinecarboxylic acid; Sigma Aldrich) in deionized water; controls were therefore prepared with the corresponding basal solvents (0.1% [v/v] DMSO and/or 1% [v/v] methanol) as appropriate.

To analyze seedling growth, root hair growth, and apical hook angle, *A*. *thaliana* (Col-0) seeds were surface sterilized for 5 min in 70% ethanol, stratified at 4°C for 48 h in the dark, and plated vertically onto half-strength Murashige and Skoog (MS) medium (Sigma Aldrich). MS medium was supplemented with relevant concentrations of additional compounds as indicated. Plants were grown under low-intensity continuous light (3 μmol m^−2^ s^−1^) at 21°C. For analysis of hypocotyl and root length, seedlings were photographed after 6 days; for analysis of root hair length and apical hook angle, seedlings were photographed after 4 days. All images were analyzed and quantified using ImageJ. An example of apical hook angle measurement is provided in Supplemental Figure S11. For CEPA (2-choloethylphosphonic acid) treatment of *Arabidopsis*, 10 μL 500 mM stock was placed topically and spread evenly onto agar plates before seed plating. Plates were wrapped with parafilm to ensure airtightness.

### Ethylene measurements in *Arabidopsis*

To quantify ethylene emission, 40 *Arabidopsis* Col-0 seeds were stratified on sterile filter paper disks in MS with 1% sucrose plates for 4 days in the dark at 4°C. Plates were transferred to 22°C in the light (16 h light:8 h dark) for 24 h. Seedlings on filter paper were then transferred to 10 mL vials sealed with a septum. To test the system, vials contained 1 mL solid MS supplemented with 1% sucrose with DMSO, 100 μM ACC, 2 mM AIB (ethylene inhibitor), and 100 μM ACC plus 10 mM of AIB. To test MyA and ETHi-74-12-16 activity, 1 mL solid MS–1% sucrose was supplemented with DMSO, 100 μM ETHi-74-12-16, or 100 μM Mya, with or without 1 μM ACC (all bottles had the same final amount of DMSO). Vials were wrapped in two layers of aluminum foil and kept at 22°C for 2 days before measurement. Three biological replicates were measured. Ethylene released in the vials was quantified using a laser-based photoacoustic spectrophotometer (ETD-300, SensorSense).

Accession numbers for major genes discussed are as follows:

*D*. *discoideum* ACO (Dictybase identifier DDB_G0277497, Accession EAL68711.1), *Petunia × hybrida* ACO (PDB: 1WA6, Accession AAC37381), and *A*. *thaliana* Acc oxidase 1 (Accession NP-179549.1).

## Data and code availability

All data are supplied within the manuscript or in the supplementary information.

### Statistical analysis

Descriptions of statistical analyses are provided in the legends of the individual figures.

## Funding

G.H.H. was supported by a PhD studentship funded by 10.13039/501100000268BBSRC DTP iCASE in collaboration with Syngenta Ltd. The CRISPR plasmids were kindly supplied by Dr. Yoichiro Kamimura, RIKEN Cell Signaling Dynamics Team, 10.13039/501100022577Center for Biosystems Dynamics Research, RIKEN(G90426). Compounds described in the manuscript have been protected through patent application. One author is employed by industry (Syngenta, K.H.).

## Author contributions

G.H.H. and R.S.B.W. designed the study, and G.H.H. carried out *D*. *discoideum* and seedling experiments. K.N., G.L., and K.H. carried out and supervised the seed germination work. C.R.L.T. and R.B. carried out the REMI-seq analysis. F.R. and G.H.H. carried out the ethylene measurement experiments. D.A. and E.R. conducted ethylene measurement work in *Arabidopsis*. R.S.B.W. conceived the project, supervised the work, and with G.H.H. wrote the paper. All authors contributed to and have approved the final manuscript.

## Supplemental information

Document S1. Supplemental Figures 1–14

Document S2. Article plus supplemental information
