# Supplementary material for: Functional mechanism study of the allelochemical myrigalone A identifies a group of ultrapotent inhibitors of ethylene biosynthesis in plants
Source: Plant Commun. 2024 Mar 8;5(6):100846. doi: 10.1016/j.xplc.2024.100846 (PMC11211550; doi:10.1016/j.xplc.2024.100846)
Supplement: Document S1. Supplemental Figures 1–14 [file mmc1.pdf]

Plant Communications, Volume *5*

Supplemental information

Functional mechanism study of the allelochemical myrigalone A identifies a group of ultrapotent inhibitors of ethylene biosynthesis in plants

George Heslop-Harrison, Kazumi Nakabayashi, Ana Espinosa-Ruiz, Francesca Robertson, Robert Baines, Christopher R.L. Thompson, Katrin Hermann, David Alabadí, Gerhard Leubner-Metzger, and Robin S.B. Williams

Supplementary Information

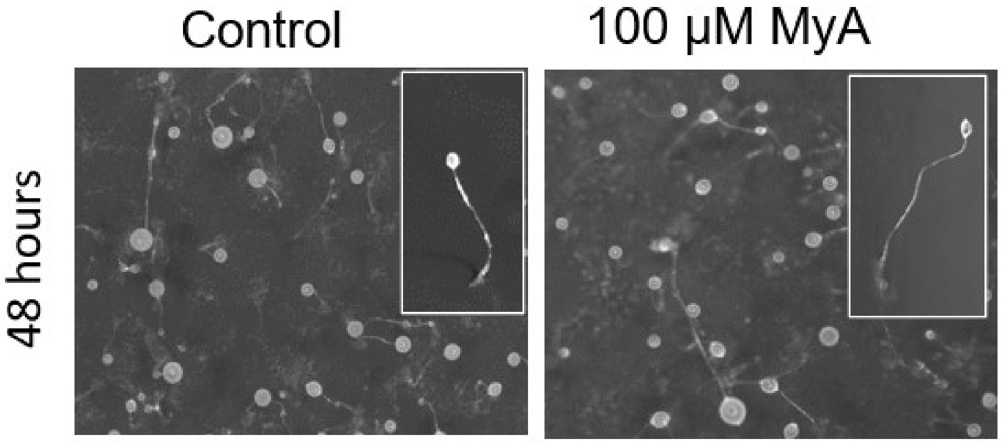

Supplementary Figure S1: MyA-treatment delays Dictyostelium development. Developmental phenotypes, in the absence of MyA (control), showing fruiting body morphology at 48 hours, from top-down view and individual fruiting bodies (side view insert). Development in the presence of MyA (100 µM) was restored at 48 hours.

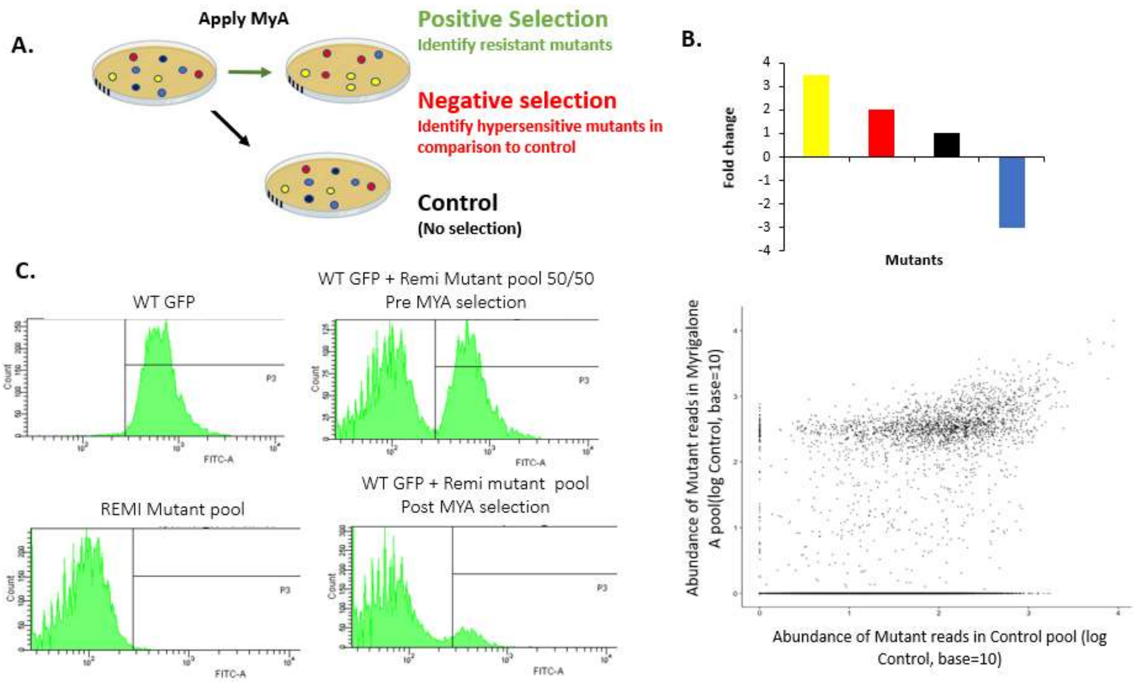

Supplementary Figure S2: MyA growth resistance screen. *(A) D. discoideum growth resistance screens are commonly used to provide an unbiased search for genes controlling sensitivity to bioactive compounds. Here, different mutants are represented by different colours. Following exposure to a compound that reduces cell growth, two mutants (coloured red and yellow) continue growth, suggesting genes lost in these mutants regulate to sensitivity. (B) For each mutant, after compound exposure, fold change in levels of each mutant identified through next generation REMI-sequencing (REMI seq) (Gruenheit et al., 2019) provides a readout for resistance or sensitivity. (C) Following the third round of screening, a resistance check was performed using 5 x 10^5 cells per ml of WT GFP expressing cells grown together with 5 x 10^5 cells from the final round of screening for 48 hours in 100 µM of MyA. The ratio of WT GFP expressing cells to REMI-seq cells was calculated using a BD FACSCanto cell analyzer. A decrease in WT-GFP cells and an increase in REMI cells shows the presence of resistant mutants. (D) Mutant sequencing read counts which correlates towards mutant abundance was plotted for MyA treatment against control pools. Those mutants which showed a significant increase in growth against control pools were deemed to show selective resistance to MyA. This supplementary figure refers to main Figure 2.*

Resistant and hyper sensitive mutants identified in Myrigalone A growth screen

D. discoideum Accession	D. discoideum Name	A. thaliana Homologue	A. thaliana Accession
Resistant Mutants			
DDB_G0272502	DDB_G0272502	N/A	N/A
DDB_G0267952	DDB_G0267952	protein disulfide-isomerase 5-1	XP_020870842.1
DDB_G0276245	acyl-CoA oxidase	acyl-coenzyme A oxidase 2, peroxisomal	XP_002866650.1
DDB_G0269448	DDB_G0269448	putative myotubularin	AAG51396.1
DDB_G0284565	ANK_REP_REGION domain-containing protein	Ankyrin repeat family protein	NP_178442.2
DDB_G0271766	pARTg	unnamed protein product	CAA0275201.1
DDB_G0270002	DDB_G0270002	kinesin-like protein KIN-4C	XP_002868397.1
DDB_G0290635	DDB_G0290635	N/A	N/A
DDB_G0278769	cstf1	Transducin/WD40 repeat-like superfamily protein	NP_974972.1
DDB_G0283633	Major facilitator superfamily domain-containing protein 5 (	molybdate-anion transporter	XP_020890378.1
DDB_G0289183	MTO1 homolog, mitochondrial	glucose-inhibited division family A protein	NP_178974.1
DDB_G0287667	DDB_G0287667	receptor-like kinase 902	NP_566589.1
DDB_G0272298	DDB_G0272298	ervatamin-B	XP_002877667.1
DDB_G0273593	DDB_G0273593	N/A	N/A
DDB_G0290743	cyp514A1	cytochrome P450 82G1	XP_002883585.1
DDB_G0287679	TTRAY2	Transducin/WD40 repeat-like superfamily protein	NP_197859.4
DDB_G0275225	FKBP12	dihydroflavonol reductase	CAP08819.1
DDB_G0277497	aco	2-oxoglutarate (2OG) and Fe(II)-dependent oxygenase superfamily protein	NP_001030834.1
DDB_G0268210	BB_PF domain-containing protein	N/A	N/A
DDB_G0270848	DDB_G0270848_ps	N/A	N/A
DDB_G0268986	TRE3-B ORF2	N/A	N/A
DDB_G0267848	beta-lactamase-type transpeptidase fold containing protein	SRKp	AJP61150.1
DDB_G0288003	EGF-like domain-containing protein	N/A	N/A
DDB_G0285385	DDB_G0285385	N/A	N/A
DDB_G0290079	pgtB / putative glycosyltransferas	sulfoquinovosyldiacylglycerol 2	NP_568085.2
DDB_G0286871	GCN5-related N-acetyltransferase	N/A	N/A
DDB_G0290627	DDB_G0290627	N/A	N/A
DDB_G0290833	rabK1 / Rab GTPase	RAB GTPase homolog 7A	NP_565521.1
DDB_G0280307	beta-lactamase family protein	hypothetical protein AXX17_ATUG03410	OAO89193.1
DDB_G0286981	DDB_G0286981	N/A	N/A
DDB_G0272336	DDB_G0272336_ps	unnamed protein product	CAA0370672.1
DDB_G0269892	DDB_G0269892	N/A	N/A
DDB_G0276057	UBiquitin regulatory X	Chain A, Thioredoxin h1	1XFL_A
DDB_G0275399	DDB_G0275399	hypothetical protein AXX17_AT3G49740	OAP03484.1
DDB_G0288061	ATP-gated ion channel P2XE	N/A	N/A
DDB_G0277145	pkaD	serine/threonine-protein kinase AtPK2/AtPK19	XP_020888566.1
DDB_G0282133	DDB_G0282133	hypothetical protein AXX17_AT5G27870	OAO90000.1
DDB_G0281915	transmembrane protein	N/A	N/A
DDB_G0274885	Cell Division Cycle 73	PHP, partial	AIU48804.1
DDB_G0285599	mcfB	adenine nucleotide transporter 1	NP_192019.1
DDB_G0269620	DDB_G0269620	N/A	N/A
DDB_G0277441	rabJ	Ras small GTP-binding family protein	NP_199326.1

D. discoideum Accession	D. discoideum Name	A. thaliana Homologue	A. thaliana Accession
DDB_G0285031	DDB_G0285031	N/A	N/A
DDB_G0281349	PH domain-containing protein	N/A	N/A
DDB_G0283373	Sulfhydryl oxidase	N/A	N/A
DDB_G0275155	DDB_G0275155	N/A	N/A
DDB_G0271700	thioredoxin-like protein	protein disulfide-isomerase 5-1	XP_020870842.1
DDB_G0288573	DDB_G0288573	N/A	N/A
DDB_G0285201	Late secretory pathway protein AVL9 homolog	N/A	N/A
DDB_G0285271	DDB_G0285271	metalloendopeptidase / zinc ion binding protein	NP_001190451.1
DDB_G0284853	DDB_G0284853	Aminotransferase-like, plant mobile domain family protein	NP_193340.2
DDB_G0284279	DDB_G0284279_ps	N/A	N/A
DDB_G0281087	gtaV	DNA-binding protein with MIZ/SP-RING zinc finger, PHD-finger and SAP domain-containing protein	NP_001032108.1
DDB_G0267492	DDB_G0267492	ATP-dependent zinc metalloprotease FTSH 4	XP_002880747.1
DDB_G0274045	3B-2	N/A	N/A
DDB_G0273543	DDB_G0273543	N/A	N/A
DDB_G0268138	putative glutathione S-transferase alpha-1	GSTF11	OAP02577.1
DDB_G0286295	DDB_G0286295	N/A	N/A
DDB_G0275615	DDB_G0275615_ps	N/A	N/A
DDB_G0293854	DDB_G0293854	N/A	N/A
DDB_G0289617	DDB_G0289617	N/A	N/A
DDB_G0291664	putative protein kinase	Mitogen activated protein kinase kinase kinase-like protein [Arabidopsis thaliana]	NP_567072.1
DDB_G0287565	DDB_G0287565_ps	N/A	N/A
DDB_G0290273	DDB_G0290273	N/A	N/A
DDB_G0290709	pks32	Chain A, 3-OXOACYL CARRIER PROTEIN SYNTHASE	1W0I_A
DDB_G0291758	DDB_G0291758	N/A	N/A
DDB_G0270730	fslB	N/A	N/A
DDB_G0273951	DDB_G0273951	expressed protein	EFH43206.1
DDB_G0277843	DG1105	N/A	N/A
DDB_G0268290	DDB_G0268290	uncharacterized protein LOC110224440	XP_020866161.1
DDB_G0291123	GLycogen Phosphorylase	alpha-glucan phosphorylase 2	NP_190281.1
DDB_G0295773	DDB_G0295773	DUF1077 family protein	EEU04121.1
DDB_G0278677	DDB_G0278677	N/A	N/A
DDB_G0273005	DDB_G0273005	N/A	N/A
DDB_G0269132	ecmB	N/A	N/A
DDB_G0276919	PhospoLipase D, GPI-specific	N/A	N/A
DDB_G0285347	DDB_G0285347	N/A	N/A
DDB_G0272304	aspS2	N/A	N/A
DDB_G0279431	DDB_G0279431	N/A	N/A
DDB_G0281767	DDB_G0281767	Regulator of chromosome condensation (RCC1) family protein [Arabidopsis thaliana]	NP_186900.3
DDB_G0277577	DDB_G0277577	E3 ubiquitin-protein ligase dbl4	XP_002880863.1
DDB_G0292560	racJ	ROP9 [Arabidopsis thaliana]	OAO98596.1
DDB_G0284683	DDB_G0284683	N/A	N/A

D. discoideum Accession	D. discoideum Name	A. thaliana Homologue	A. thaliana Accession
DDB_G0292156	fscG	N/A	N/A
DDB_G0276527	DDB_G0276527	N/A	N/A
DDB_G0284511	DDB_G0284511	N/A	N/A
DDB_G0279679	DDB_G0279679	N/A	N/A
DDB_G0292696	colA	N/A	N/A
DDB_G0267602	DDB_G0267602	Regulator of chromosome condensation (RCC1) family protein [Arabidopsis thaliana]	NP_201191.1
DDB_G0283467	DDB_G0283467	N/A	N/A
DDB_G0285299	DDB_G0285299	Protein kinase superfamily protein [Arabidopsis thaliana]	NP_188511.1
DDB_G0290689	DDB_G0290689	diadenosine 5',5'''-P1,P4-tetraphosphate hydrolase, putative [Arabidopsis thaliana]	AAF76368.1
DDB_G0269222	gefB	N/A	N/A
DDB_G0287013	DDB_G0287013	phytochrome and flowering time regulatory protein (PFT1) [Arabidopsis thaliana]	NP_001077596.1
DDB_G0282291	potA	N/A	N/A
DDB_G0288239	sibE	N/A	N/A
DDB_G0268512	wrn	DNA helicase (Recql4A)	NP_172562.2
DDB_G0280171	psiP	N/A	N/A
DDB_G0275023	act22	actin-11	NP_187818.1
DDB_G0273545	nfyC-2	nuclear factor Y, subunit C1	NP_190428.1
DDB_G0287501	DDB_G0287501	Jojoba acyl CoA reductase-related male sterility protein	NP_187805.1
DDB_G0276823	DDB_G0276823	N/A	N/A
DDB_G0269278	DDB_G0269278	DECAPPING NUCLEASE DXO1	6DKN_A
DDB_G0279573	DDB_G0279573	TOM3	OAP08400.1
DDB_G0271286	clasp	CLIP-associated protein	NP_849997.2
DDB_G0293542	DDB_G0293542	phytanoyl-CoA dioxygenase (PhyH) family protein	NP_001325094.1
DDB_G0281567	fhkC	SNF1 family protein kinase, partia	AAD00542.1
DDB_G0280155	DDB_G0280155_ps	N/A	N/A
DDB_G0278863	fray1	protein kinase-like protein	CAC01871.1
DDB_G0277055	DDB_G0277055	N/A	N/A
DDB_G0273623	hspE-2	heat shock protein 70B	NP_173055.1
DDB_G0277301	DDB_G0277301	N/A	N/A
DDB_G0277597	gpt3	N/A	N/A
DDB_G0290125	DDB_G0290125	N/A	N/A
DDB_G0268212	DDB_G0290125	RNA helicase - like protein	CAA16726.1

D. discoideum Accession	D. discoideum Name	A. thaliana Homologue	A. thaliana Accession
Hypersensitive mutants			
DDB_G0292386	carmil	N/A	N/A
DDB_G0268248	DDB_G0268248	N/A	N/A
DDB_G0288695	DDB_G0288695	N/A	N/A
DDB_G0274597	ctnA	N/A	N/A
DDB_G0269108	catB	CAT2	OAO97606.1
DDB_G0289031	DDB_G0289031	alpha/beta-Hydrolases superfamily protein	NP_192960.1
DDB_G0273949	DDB_G0273949	AT5G41080	BAH57127.1
DDB_G0274279	tssc1	Transducin/WD40 repeat-like superfamily protein	NP_173478.2

Supplementary Figure S3: MyA resistance mutants identified in growth resistance screen. *Following MyA growth resistance screen, 125 mutants were identified showing enhanced growth (green, resistant) or reduced growth (red, sensitive), shown here with Dictybase gene ID, gene name (if known), GO terminology, and potential A. thaliana homologue and accession number. This supplementary figure refers to main Figure 2.*

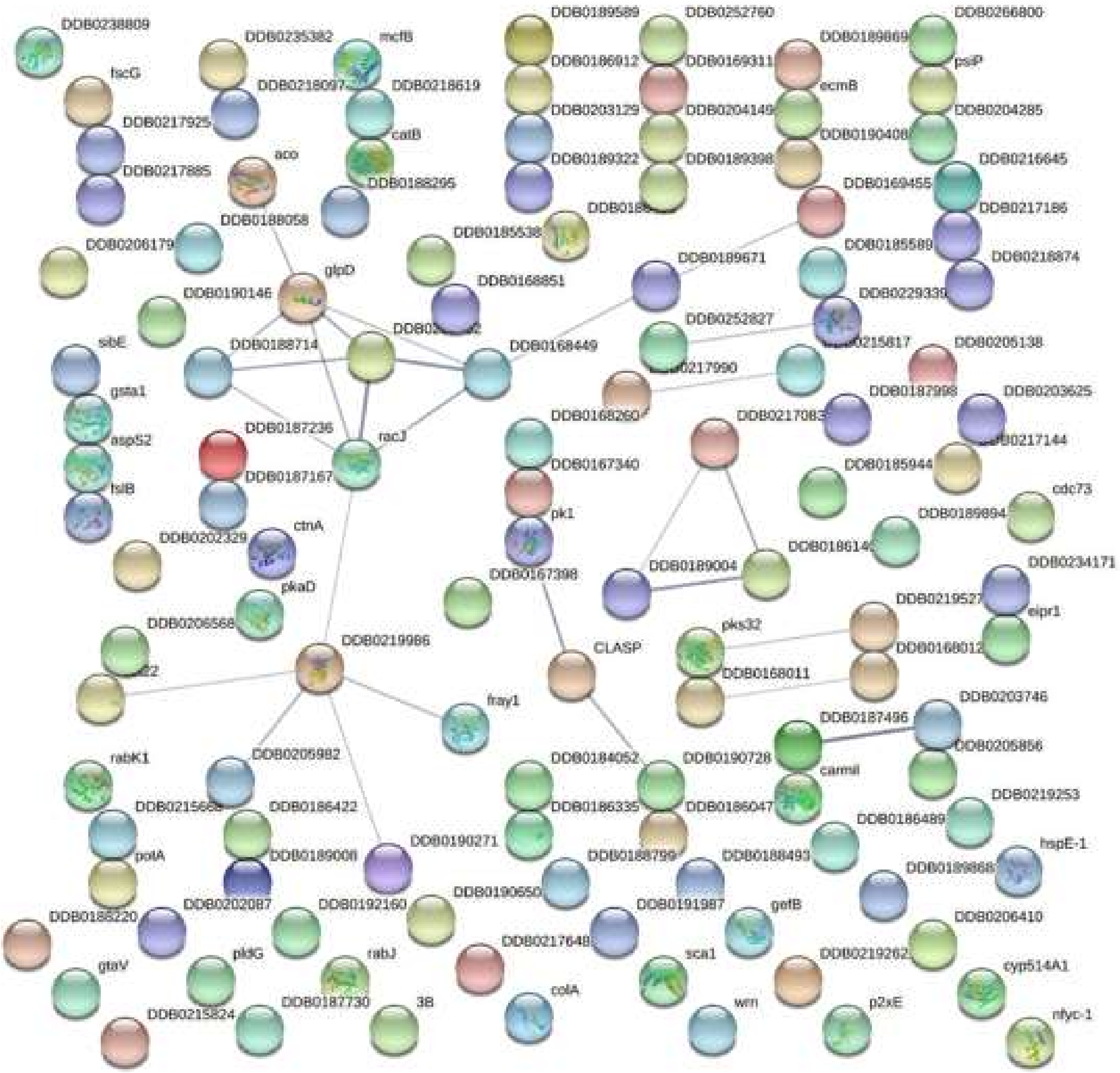

Supplementary Figure S4: String analysis of all MyA resistant mutants. *String Analysis for all proteins ablated in 112 MyA resistant mutants identified during mutant library screening. Lines connecting individual mutants indicate connectivity. This supplementary figure refers to main Figure 2.*

A

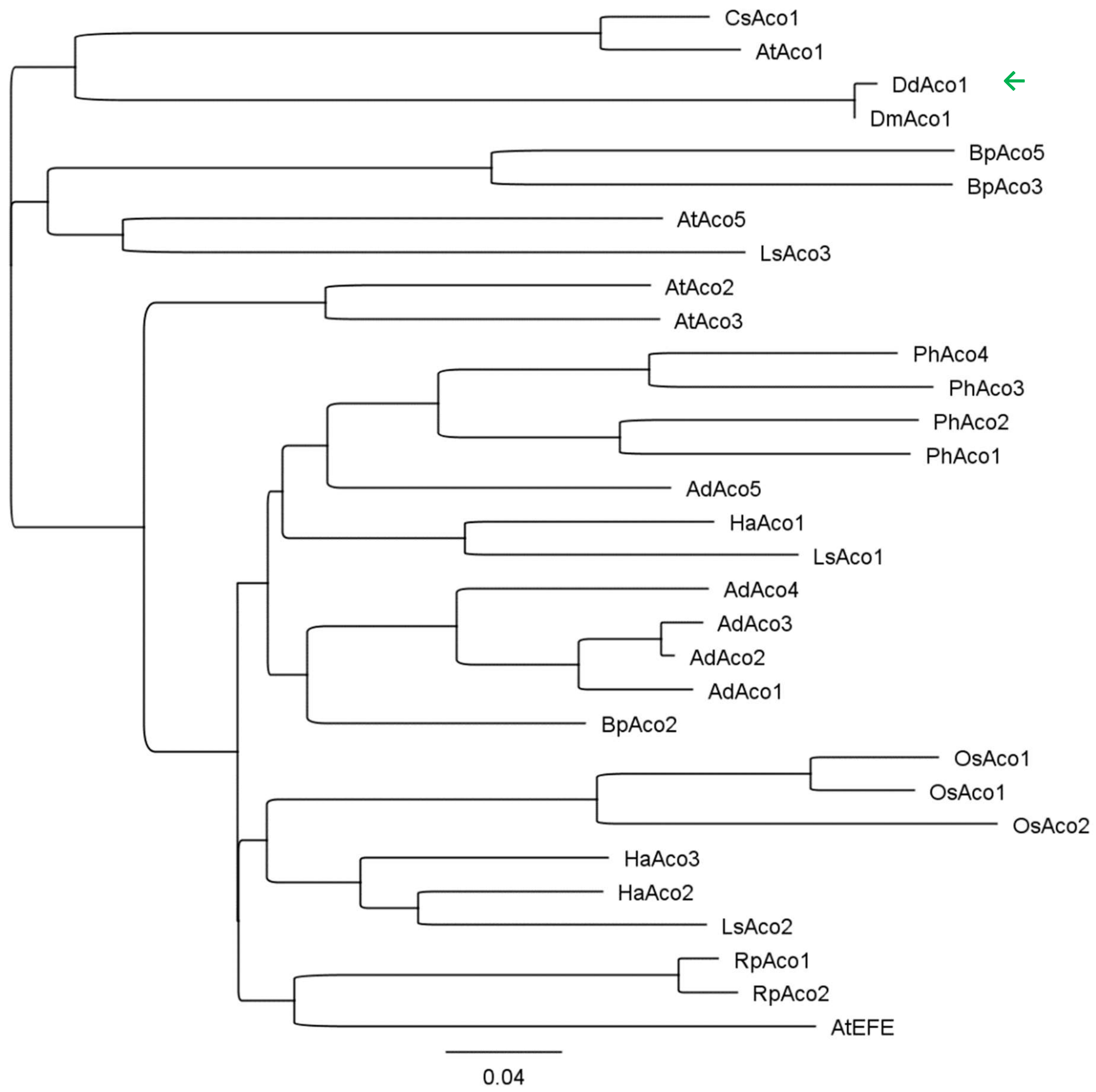
CsAco1
AtAco1
DdAco1
DmAco1
BpAco5
BpAco3
AtAco5
LsAco3
AtAco2
AtAco3
PhAco4
PhAco3
PhAco2
PhAco1
AdAco5
HaAco1
LsAco1
AdAco4
AdAco3
AdAco2
AdAco1
BpAco2
OsAco1
OsAco1
OsAco2
HaAco3
HaAco2
LsAco2
RpAco1
RpAco2
AtEFE
0.04

B

Name	Accession	Description	Organism
ACTACCOXI	M97961.1	AdAco1	Actinidia deliciosa
HQ293205	HQ293205.1	AdAco2	Actinidia deliciosa
HQ293207	HQ293207.1	AdAco3	Actinidia deliciosa
HQ293209	HQ293209.1	AdAco4	Actinidia deliciosa
HQ293211	HQ293211.1	AdAco5	Actinidia deliciosa
NM_127517	NM_127517.5	AtAco1	Arabidopsis thaliana
NM_104918	NM_104918.5	AtAco2	Arabidopsis thaliana
NM_101073	NM_101073.3	AtAco3	Arabidopsis thaliana
NM_106382	NM_106382.3	AtAco5	Arabidopsis thaliana
NM_100380	NM_100380.4	AtEFE	Arabidopsis thaliana
AY154649	AY154649.1	BpAco2	Betula pendula
X97992	X97992.1	BpAco3	Betula pendula
X97994	X97994.1	BpAco5	Betula pendula
XM_010490852	XM_010490852.2	CsAco1	Camelina sativa
XM_637519	XM_637519.1	DdAco1	Dictyostelium discoideum AX4
AB291210	AB291210.1	DmAco1	Dictyostelium mucoroides
HNNACC	L29405.1	HaAco1	Helianthus annuus
HAU62554	U62554.1	HaAco2	Helianthus annuus
HAU62555	U62555.1	HaAco3	Helianthus annuus
AB158345	AB158345.1	LsAco1	Lactuca sativa
AB158346	AB158346.1	LsAco2	Lactuca sativa
AB158347	AB158347.1	LsAco3	Lactuca sativa
AF049888	AF049888.1	OsAco1	Oryza sativa
X85747	X85747.1	OsAco1	Oryza sativa Indica Group
AF049889	AF049889.1	OsAco2	Oryza sativa
PETACO1A	L21976.2	PhAco1	Petunia x hybrida
PETACO2A	L21977.1	PhAco2	Petunia x hybrida
PETACO3A	L21978.1	PhAco3	Petunia x hybrida
PETACO4A	L21979.1	PhAco4	Petunia x hybrida
RpACO1	Y10034.1	RpAco1	Rumex palustris
AF041479	AF041479.1	RpAco2	Rumex palustris

Supplementary Figure S5. Phylogenetic analysis of Dictyostelium and plant ACO proteins. ***(A)*** *Neighbor-joining phylogentic tree is based on similarity of proteins. Alignment of sequences and neighbough joining tree was constructed in Geneious R.9 using Jukes-Cantor as the genetic distance model. Scale bar indicates bootstrap values (100 replications).* ***(B)*** *Protein sequences used for phylogenetic analysis.*

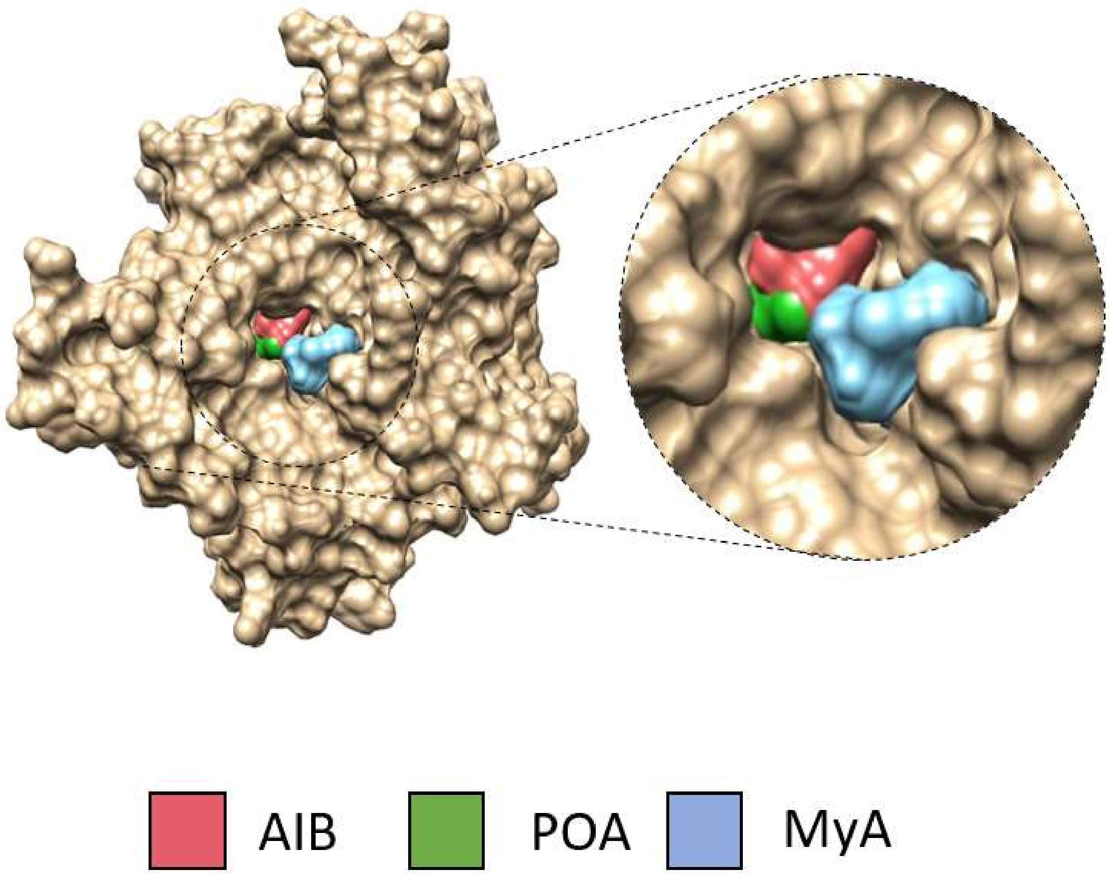

Supplementary Figure S6. Tertiary structure analysis of D. discoideum ACO enzymes and MyA binding. *Space-filling model of D. discoideum ACO enzyme, shown with binding by existing ACO inhibitors AIB (pink), POA (green) and MyA (light blue) within the catalytic pocket.*

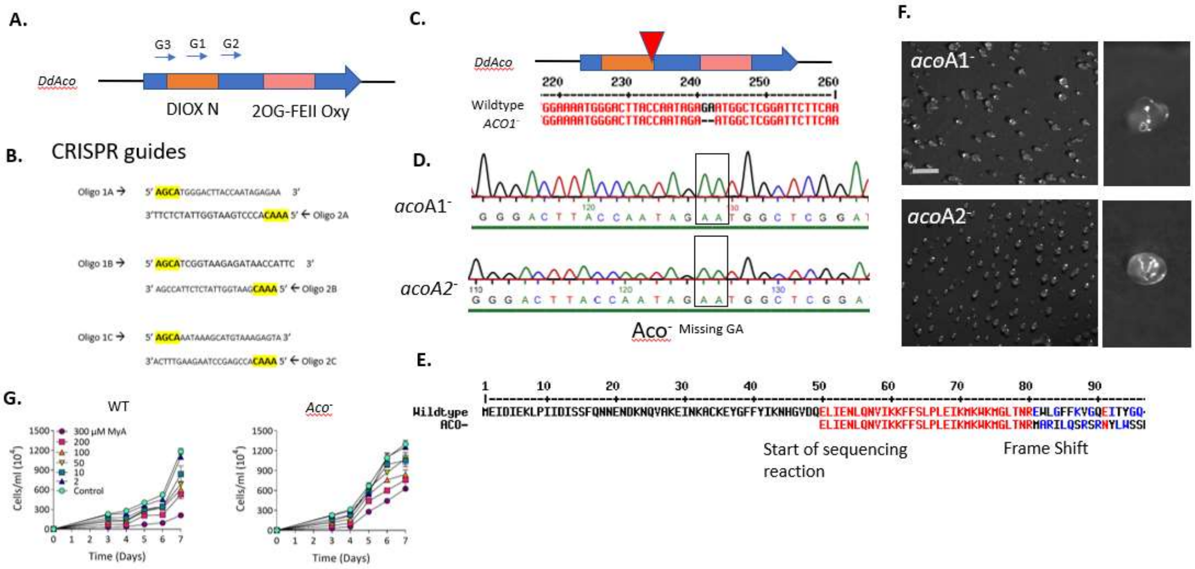

Supplementary Figure S7: Genetic ablation of D. discoideum acoA gene. *(A) Schematic of Dictyostelium ACO gene showing location of Crispr guides and (B) Crispr guide sequences. (C, D) Schematic showing mutated site (red triangle) and sequence analysis for two independent mutations, leading to (E) a frame shift from AA80. (F) Both independent mutants show common block in development at the mound stage (size bar = 1mm).* (G) *Growth sensitivity of Wild type (Ax3) and Aco- cells were analysed as described in the methods. Growth rates were calculated, for each MyA concentration in cell type, and used to provide the secondary plot shown in Figure 3. Data represents 3 biological repeats analysed with 3 technical repeats. This supplementary figure refers to main Figure 2.*

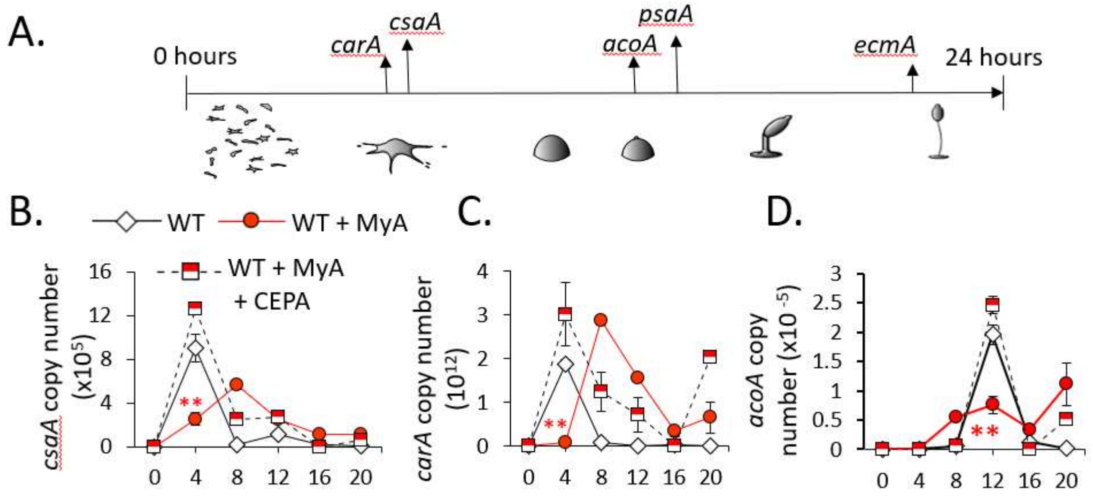

Supplementary Figure S8: **MyA regulates *D. discoideum* development through loss of ethylene.** (A) To quantify developmental effects of MyA, expression of specific developmental genes can be monitored, including (B) csA (Contact site A), (C) cAR1 (cAMP receptor 1) and (D) AcoA. Here, wild type cells, in solvent only conditions, or with MyA (100 µM) alone or in the presence of CEPA, were allowed to develop for the indicated time periods, RNA was extracted, and gene expression determined using quantitative PCR, expressed as absolute copy number. Data is derived from 3 independent experiments analysed in triplicate, and shown as mean ± SEM, with statistical analysis performed using a Student's *t*-test comparing WT with WT + MyA with ** (red) P<0.01, and WT + Mya with WT + MyA + CEPA ** (black) P< 0.01 and ***P<0.001 at the point of greatest difference.

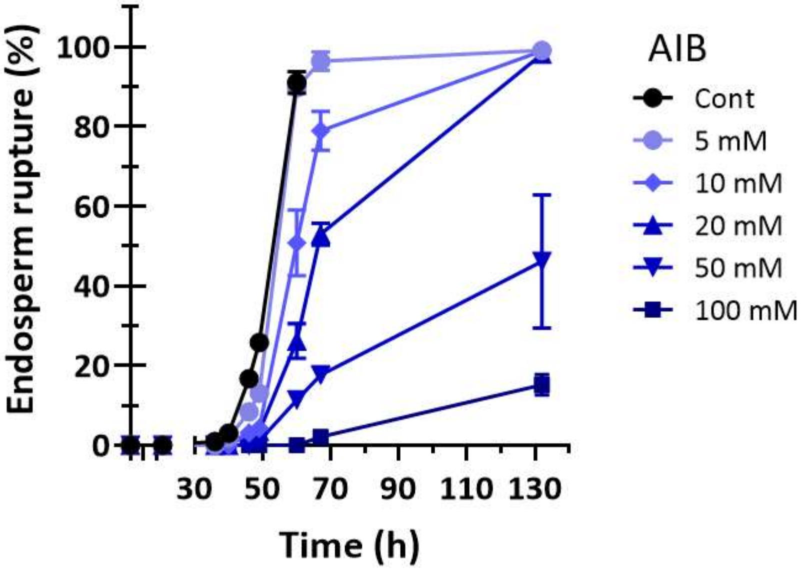

Supplementary Figure S9: Dose dependent reduction in A. thaliana endosperm rupture following ethylene inhibition by AIB. *This supplementary figure refers to main Figure 5.*

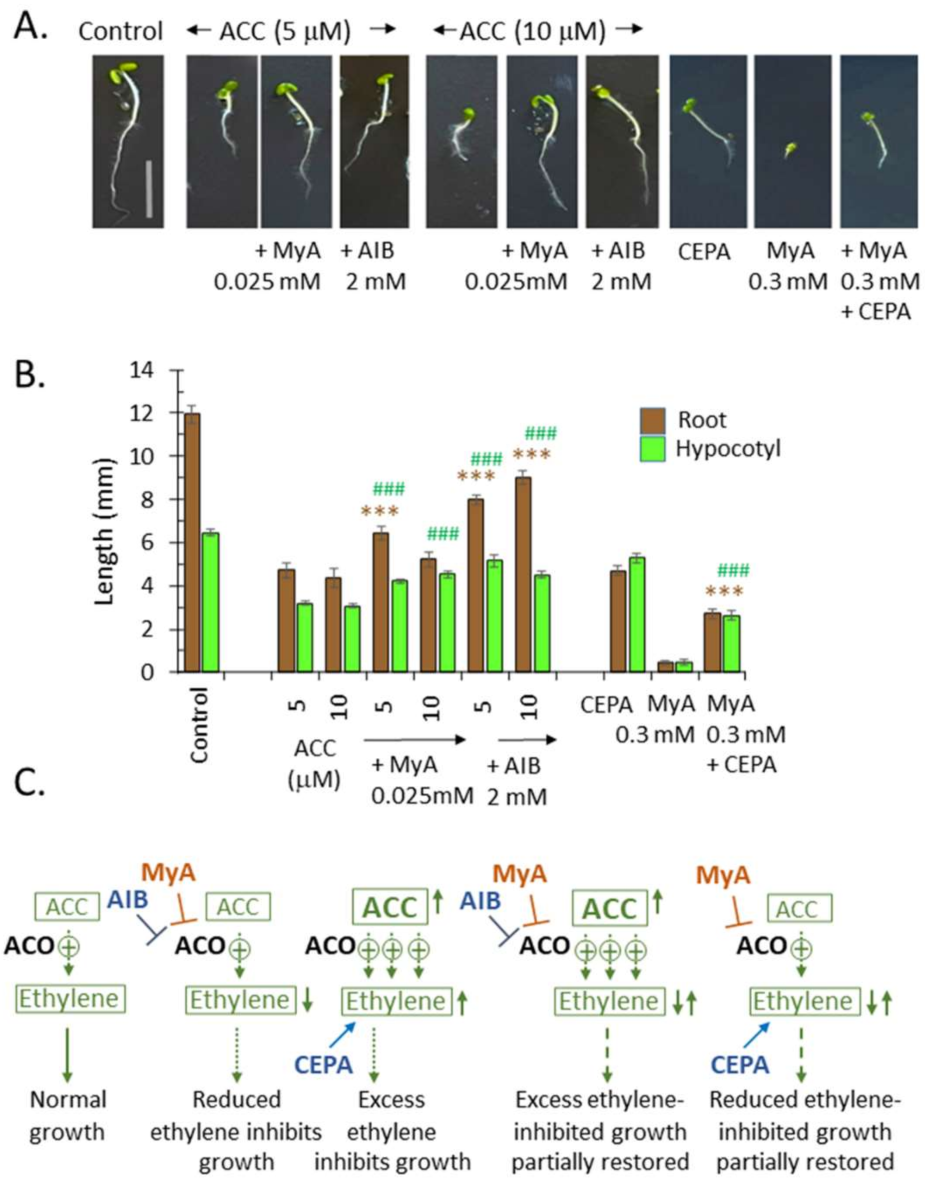

***Supplementary Figure S10:* MyA inhibits ethylene response in *A. thaliana* seedlings.** (A, B) To provide further evidence for the effect of MyA on inhibition of ethylene production in *A. thaliana* Col-0 seedlings, root and hypocotyl growth was assess in the presence of increased ethylene by addition of ACC (5 and 10 μM) and with the addition of MyA (25 µM) or AIB (2mM), or through CEPA treatment ± MyA (300 μM)(n=3). Statistical analysis is shown for root (brown asterisk) and hypocotyl (green hash) comparing ACC with corresponding MyA or AIB treatments or comparing MyA with MyA and CEPA. (C) Schematic showing the effects of MyA on *A. thaliana* seedling growth, reducing effects of ACC-dependent ethylene production by ACO, and reducing seedling growth with effects partially rescued by exogenous ethylene provided by CEPA. Statistical analysis performed using a Student's *t*-test for control and the lowest concentration of compounds showing an effect (brown asterisk for root, green hatch for hypocotyl) *** or $^{###}$P<0.001. Data derived from three independent experiments, with at least 5 seedlings per experiments shown as mean ± SEM.

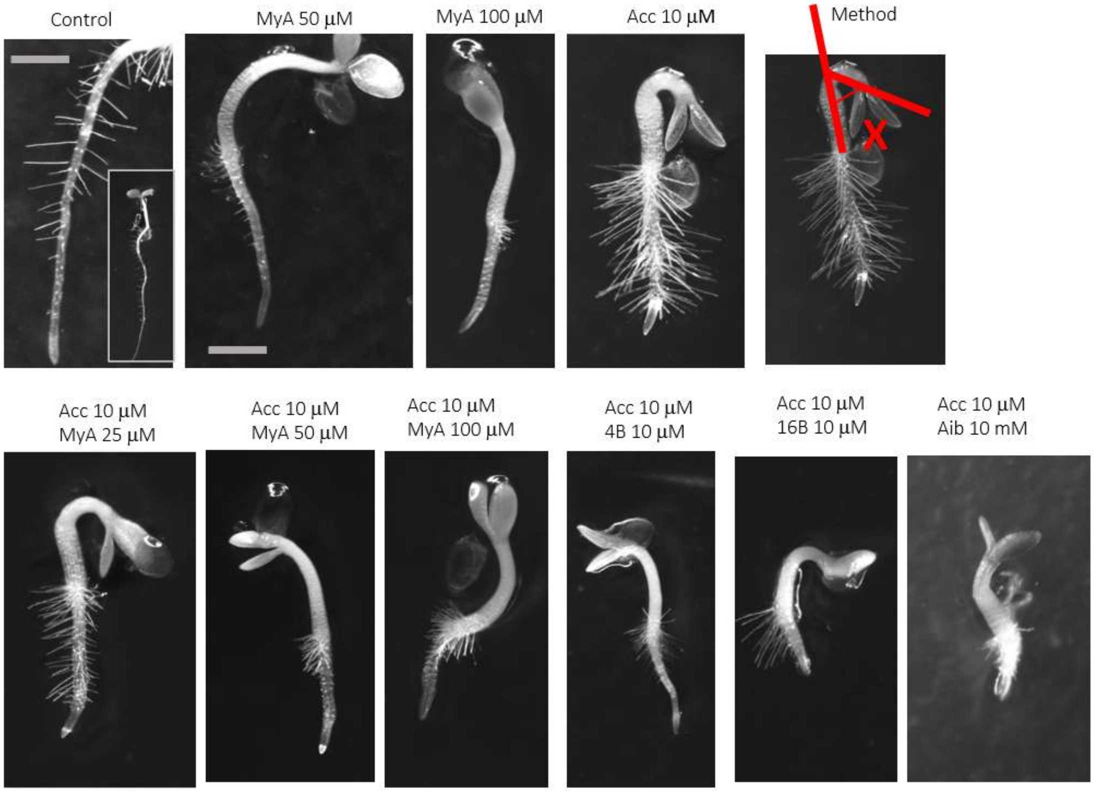

Supplementary Figure S11: MyA blocks ethylene promoted Apical Hook curvature. *MyA, 4B and 16B treatment of A. thaliana seedlings was assessed for the inhibition of apical hook curvature as part of the ethylene triple response after 4 days of growth. MyA caused a concentration dependant decrease in the bothACC promoted ACC induced apical hook curvature. Images shows representative seedling from 3 independent experiments, each with 5 seedlings. Ethylene response can be seen for ACC treatment, with increased root hair density and length together with an exaggerated apical hook. Scale bar = 0.3 mm. Data represents average and SEM (error bars) shown with statistical analysis performed using a student t test. *** Indicate P<0.005). Statistical analysis is performed for Acc treatment against Acc plus MyA, 4B and 16B treatment. Apical hook angle was measured as Angle X shown in (A).*

.

		Smiles	Pubchem ID	Cluster in binding pocket	Full fitness (Kcal/mol)	Estimated ΔG (kcal/mol)
MyA		CC1C(=O)C(C(=O)C(C1=O)(C)C)C(=O)CCC2=CC=CC=C2	11722329	0	-2016	-7.21
POA		C1=CN=C(C=N1)C(=O)O	1047	0	-1993	-6.35
2-AIB		CC(C)(C(=O)O)N	6119	0	-1946	-5.96
ETHi-1	1	[Na+].COC(=O)C1C(CC(=O)C=C1[O-])c2ccccc2	70161725	0	-1973	-6.23
ETHi-2	2	CN(C)CCO.CN1C(=O)C(C(=O)Nc2ccc(Cl)c(Cl)c2)C(=O)N(C)C1=O	179008	0	-2101	-7.54
ETHi-3	3	Oc1ccc(cc1)C(=O)OCc2ccccc2	7180	0	-1952	-7.58
ETHi-4	4	O=C(OCc1ccccc1)ON2C(=O)CCC2=O	83172	0	-1972	-6.90
ETHi-5	5	CCOC(=O)C1C(CC(=O)CC1=O)c2ccccc2	12617805	0	-2003	-6.57
ETHi-6	6	O=C(Nc1ccccc1)C2C(=O)NC(=O)NC2=O	10776860	2	-2112	-6.78
ETHi-7	7	CN1C(=O)C(C(=O)Nc2ccccc2)C(=O)N(C)C1=O	71685841	0	-2100	-6.87
ETHi-8	8	CCCCNC(=O)C1C(=O)N(C)C(=O)N(C)C1=O	75391698	0	-2123	-7.08
ETHi-9	9	CN1C(=O)C(C(=O)c2cccc(c2)C(F)(F)F)C(=O)N(C)C1=O	4323130	0	-2050	-6.89
ETHi-10	10	COC(=O)C1C(C)CC(=O)CC1=O	4661376	2	-2004	-6.26
ETHi-11	11	[I-].Oc1ccc(C(=O)C[n+]2ccccc2)c(O)c1	459585	1	-1954	-6.02
ETHi-12	12	Oc1ccc(C(=O)Cc2ccccc2)c(O)c1	138005	0	-1955	-7.21
ETHi-13	13	CC1=CC(=O)C(C(=O)C=Cc2ccc(C)cc2)C(=O)O1	74862511	0	-1996	-7.39
ETHi-14	14	COc1ccccc1C(=O)N(C)Cc2ccccc2	4366209		-1940	-7.11
ETHi-15	15	C\C(=N/Nc1ccccc1)\C2C(=O)OC(=CC2=O)C	154733991	0	-1972	-7.17
ETHi-16	16	O=C(OCc1ccccc1)C2CCCCC2=O	519917	0	-1993	-7.26
ETHi-17	17	CN1C(=O)C(C(=O)c2ccc(Cl)cc2)C(=O)N(C)C1=O	2785882		-2065	-6.96
ETHi-18	18	CCOC(=O)C(=O)C1C(=O)C(C)C(=O)C1=O	240733	0	-2000	-6.26
ETHi-19	19	COc1cc(O)c(C(=O)CCc2ccccc2)c(OC)c1	270058	0	-1947	-6.80
ETHi-20	20	O=C(\C=C\c1ccccc1)C2C(=O)c3ccccc3C2=O	948573	0	-1965	-7.56
ETHi-21	21	CN1C(=O)C(C(=O)CC(=O)C)C(=O)N(C)C1=O	1487846	0	-2108	-7.21
ETHi-22	22	COc1cc(O)c(C(=O)C=Cc2ccc(C)cc2)c(OC)c1	3932496	0	-1937	-6.88
ETHi-23	23	COC(=O)C1CN(CCC1=O)C(=O)c2ccccc2	12414561	0	-2000	-6.56
ETHi-24	24	CC1(C)CC(=O)C(C(=O)Cc2ccccc2)C(=O)C1	788371	0	-1991	-7.12
ETHi-25	25	COc1cc(OC)c(C(=O)\C=C\c2ccc(Cl)cc2)c(OC)c1	5961012	0	-1928	-8.13
ETHi-26	26	O=C(Cc1ccccc1)ON2C(=O)CCC2=O	13339790	0	-1957	-6.78
ETHi-27	27	O=C(CCc1ccccc1)ON2C(=O)CCC2=O	13968020	0	-1970	-6.86
ETHi-28	28	COc1cccc(OC)c1C(=O)NCc2ccc(Cl)cc2	1122127	0	-1952	-7.87
ETHi-29	29	CC1=CC(=O)C(C(=O)C=Cc2ccccc2)C(=O)O1	45835562		-1995	-7.31
ETHi-30	30	CN1C(=O)C(C(=O)Nc2ccc(Cl)c(Cl)c2)C(=O)N(C)C1=O	179008	0	-2101	-7.54
ETHi-31	31	COc1cc(OC)c(C(=O)\C=C\c2ccc(F)cc2)c(OC)c1	6082788	0	-1927	-7.18
ETHi-32	32	CC1=CC(=O)C(C(=O)\C=C\c2ccccc2)C(=O)O1	16279155	0	-1995	-7.31
ETHi-33	33	CC1CN(CC(C)O1)C(=O)CS(=O)(=O)Cc2ccc(Cl)cc2	2767203	0	-1966	-6.67
ETHi-34	34	O=C(CC1C(=O)CCCC1=O)c2ccccc2	1478699	0	-1969	-7.04
ETHi-35	35	Clc1cccc(Cl)c1OCC(=O)NN2C(=O)CSC2=S	3611702	0	-1920	-7.61
ETHi-36	36	COC(=O)C1C(CC(=O)CC1=O)c2ccccc2	2786945	0	-2002	-6.47
ETHi-37	37	Oc1cc(O)c(C(=O)CCc2ccccc2)c(O)c1	1226045	0	-1974	-6.98
ETHi-38	38	O=C(NC1CCC(=O)OC1=O)OCc2ccccc2	107506	1	-1996	-7.03
ETHi-39	39	COc1cc(OC)c(C(=O)\C=C\c2ccccc2Cl)c(OC)c1	5957910	0	-1917	-7.74
ETHi-40	40	COC(=O)C1C(=O)C(C(=O)C=Cc2occc2)C(=O)CC1(C)C	2834749	0	-2021	-7.08
ETHi-41	41	CN1C(=O)C(CC(=O)c2ccccc2)C(=O)N(C)C1=O	3800750	0	-2049	-6.91

		Smiles	Pubchem ID	Cluster in binding pocket	Full fitness (Kcal/mol)	Estimated ΔG (kcal/mol)
		MYA	11722329	0	-2016	-7.21
ETHi-42	42	CN(C)c1ccc(C=CC(=O)C2C(=O)OC(=CC2=O)C)cc1	4804033	0	-1996	-7.16
ETHi-43	43	COc1cc(O)cc(O)c1C(=O)\C=C\c2ccccc2	641785		-1949	-6.85
ETHi-44	44	COc1ccc(cc1F)C(=O)N(C)Cc2ccccc2	39963891	0	-1936	-6.89
ETHi-45	45	Cc1cccc(C)c1NS(=O)(=O)Cc2ccccc2	867535	0	-1967	-6.69
ETHi-46	46	COc1cc(O)c(C(=O)C=Cc2ccccc2)c(OC)c1	242589	0	-1940	-8.16
ETHi-47	47	COC(=O)C1CCC(=O)C(C(=O)C)C1=O	3624793	0	-2030	-6.22
ETHi-48	48	CCCC(=O)C1C(=O)CC(C)(C)C(C(=O)OC)C1=O	2834748	0	-2028	-6.67
ETHi-49	49	CSc1ccc(C=CC(=O)C2C(=O)OC(=CC2=O)C)cc1	5213076	0	-1999	-7.56
ETHi-50	50	CC(C)c1ccc(C=CC(=O)C2C(=O)OC(=CC2=O)C)cc1	73245192	0	-1994	-7.06
ETHi-51	51	Clc1ccc(C=CC(=O)Oc2ccc3OCOc3c2)cc1	4826960	1	-1938	-7.34
ETHi-52	52	CC1=CC(=O)C(C(=O)C=Cc2ccc(cc2)C(C)(C)C)C(=O)O1	4827180	0	-1988	-7.16
ETHi-53	53	CC1=CC(=O)C(C(=O)C=Cc2cccc(C)c2)C(=O)O1	3896373	0	-1995	-7.41
ETHi-54	54	COc1cccc(OC)c1OC(=O)C=Cc2oc(C)cc2	5173811	0	-1934	-7.73
ETHi-55	55	CN1C=C(C(=O)N(C)C1=O)S(=O)(=O)Oc2ccccc2	7197007	1	-2048	-7.55
ETHi-56	56	CC1=CC(=O)C(C(=O)C=Cc2ccc(OC(F)F)cc2)C(=O)O1	5205201	0	-1995	-7.24
ETHi-57	57	CN1C(=O)N(C)C(=O)N(CC(=O)N2CCCCC2)C1=O	671273	0	-2117	-7.33
ETHi-58	58	CN1C(=O)N(C)c2ncc(C)c(SCc3ccc(C)cc3)c2C1=O	7424300	0	-2028	-7.34
ETHi-59	59	CN(Cc1ccccc1C)C(=O)C2=CN(C)C(=O)C=C2	56817769	0	-1943	-7.03
ETHi-60	60	CC1=CC(=O)C(C(=O)C=Cc2ccccc2OC(F)F)C(=O)O1	4542746	0	-1988	-8.00
ETHi-61	61	COC(=O)c1ccc(C=CC(=O)C2C(=O)OC(=CC2=O)C)cc1	4034774	0	-1985	-7.50
ETHi-62	62	CN1C(=C(C(=O)CSc2nnnn2C)C(=O)N(C)C1=O)N	2109821	0	-2129	-7.59
ETHi-63	63	Clc1cccc(C=CC(=O)OC2CCCCC2=O)c1	4650586	0	-1967	-7.54
ETHi-64	64	COc1ccc(C(=O)C)c(OC(=O)C=Cc2cccs2)c1	4038300	0	-1941	-8.13
ETHi-65	65	CCN(Cc1c(F)cccc1Cl)C(=O)CN2C(=O)OC(C)(C)C2=O	72914501	0	-2009	-7.28
ETHi-66	66	CC1(C)CCC(=O)N(C1=O)c2ccc(cc2)N3CCOCC3	2809720	0	-1926	-7.08
ETHi-67	67	COC(=O)C1C(=O)C(C(=O)C)C(=O)CC1(C)C	2834747	0	-2022	-6.33
ETHi-68	68	Ic1ccc(\C=C\C(=O)C2C(=O)c3ccccc3C2=O)cc1	1116074	0	-1969	-7.82
ETHi-69	69	CN1C(=C(C(=O)CNc2ccc(Cl)c(Cl)c2)C(=O)N(C)C1=O)N	2437215	0	-2035	-8.12
ETHi-70	70	COc1ccccc1CNC(=O)CN2C(=O)OC(C)(C)C2=O	72853790	0	-1999	-7.80
ETHi-71	71	CC1=CC(=O)C(C(=O)C=Cc2ccccc2C)C(=O)O1	5023048	0	-1995	-7.52
ETHi-72	72	CC1=CC(=O)C(C(=O)C=Cc2cccc(Cl)c2)C(=O)O1	72079420	0	-2002	-7.60
ETHi-73	73	CC1=CC(=O)C(C(=O)C=Cc2ccc(C)cc2C)C(=O)O1	72079415	0	-1995	-7.48
ETHi-74	74	CC1=CC(=O)C(C(=O)C=Cc2ccc(F)c(Br)c2)C(=O)O1	72079384	0	-1996	-7.78
ETHi-75	75	CC1=CC(=O)C(C(=O)C=Cc2ccc(cc2)CN)C(=O)O1	4667031	0	-1990	-7.51
ETHi-76	76	COc1ccc(Br)cc1C=CC(=O)C2C(=O)OC(=CC2=O)C	3532217	0	-1986	-7.76
ETHi-77	77	O=C(C1CCc2occc2C1=O)c3ccccc3	2950624	0	-1959	-6.55
ETHi-78	78	O=C(C=Cc1cccs1)C2C(=O)c3ccccc3C2=O	4653743		-1977	-7.56
ETHi-79	79	COc1ccc(C=CC(=O)C2C(=O)COC2=O)cc1	3544001	0	-2001	-6.70
ETHi-80	80	CC1=CC(=O)C(C(=O)\C=C\c2ccccc2Br)C(=O)O1	5951911	0	-1991	-7.69
ETHi-81	81	COC1=CC(=O)NC(=O)N1C(=O)Cc2ccccc2	750201	0	-2025	-7.32
ETHi-82	82	COc1cccc(OC)c1C(=O)NCc2ccc(C)cc2	743463	0	-1948	-7.38
ETHi-83	83	CC1=CC(=O)C(C(=O)C=Cc2ccsc2)C(=O)O1	45835549	0	-2009	-7.22
ETHi-84	84	CC1(C)CC(=O)C(C(=O)Cc2ccccc2)C(=O)C1	788371	0	-1989	-7.25

Supplementary Figure S12: *In silico analysis of novel compounds (ETHi) binding to Petunia Aco, showing similes, Pubchem ID, binding pocket, full fitness, delta G for 83 compounds related to MyA.This supplementary figure refers to main Figure 7.*

Paper name	No.	Activity*	MolPort ID	IUPAC Name
ETHi-57-1	1	-	002-088-741	1,3-dimethyl-5-[2-oxo-2-(piperidin-1-yl)ethyl]-1,3,5-triazinane-2,4,6-trione
ETHi-28-2	2	-	001-545-115	N-[(4-chlorophenyl)methyl]-2,6-dimethoxybenzamide
ETHi-46-3	3	-	000-165-364	(2E)-1-(2-hydroxy-4,6-dimethoxyphenyl)-3-phenylprop-2-en-1-one
ETHi-21-4	4	-	002-852-891	1,3-dimethyl-5-(3-oxobutanoyl)-1,3-diazinane-2,4,6-trione
ETHi-70-5	5	-	028-724-729	2-(5,5-dimethyl-2,4-dioxo-1,3-oxazolidin-3-yl)-N-[(2-methoxyphenyl)methyl]acetamide
ETHi-68-6	6	-	001-893-172	2-[(2E)-3-(4-iodophenyl)prop-2-enoyl]-2,3-dihydro-1H-indene-1,3-dione
ETHi-77-7	7	*	000-263-283	5-benzoyl-4,5,6,7-tetrahydro-1-benzofuran-4-one
ETHi-67-8	8	*	002-137-080	methyl 5-acetyl-2,2-dimethyl-4,6-dioxocyclohexane-1-carboxylate
ETHi-62-9	9	-	004-271-067	6-amino-1,3-dimethyl-5-{2-[(1-methyl-1H-1,2,3,4-tetrazol-5-yl)sulfanyl]acetyl}-1,2,3,4-tetrahydropyrimidine-2,4-dione
ETHi-69-10	10	*	028-826-119	6-amino-5-{2-[(3,4-dichlorophenyl)amino]acetyl}-1,3-dimethyl-1,2,3,4-tetrahydropyrimidine-2,4-dione
ETHi-54-11	11	**	044-278-648	2,6-dimethoxyphenyl 3-(5-methylfuran-2-yl)prop-2-enoate
ETHi-74-12	12	***	044-251-366	3-[3-(3-bromo-4-fluorophenyl)prop-2-enoyl]-6-methyl-3,4-dihydro-2H-pyran-2,4-dione
ETHi-72-13	13	ND	044-250-800	3-[3-(3-chlorophenyl)prop-2-enoyl]-6-methyl-3,4-dihydro-2H-pyran-2,4-dione
ETHi-25-14	14	-	000-658-456	(2E)-3-(4-chlorophenyl)-1-(2,4,6-trimethoxyphenyl)prop-2-en-1-one
ETHi-39-15	15	ND	003-803-656	(2E)-3-(2-chlorophenyl)-1-(2,4,6-trimethoxyphenyl)prop-2-en-1-one
ETHi-84-16	16	***	002-137-086	5,5-dimethyl-2-(2-phenylacetyl)cyclohexane-1,3-dione

Supplementary Figure S13: *Bioactivity analysis of novel ethylene inhibitors (ETHi) for efficacy in reducing the phenotype identified in blocking ethylene production in D. discoideum. Compounds predicted by molecular modelling to bind to the plant ACO enzyme (Supplementary. Fig. 7) were tested for developmental delay effects in D. discoideum, to mimic that shown for established ethylene inhibitors and MyA, blocking development at the mound stage, where '***' = delayed to mounds (MyA-like) , '**' = mostly mounds several breakthrough, '*' = less delayed development and '-' = no effect on development. Compounds are listed as preliminary names, activity related to inhibition of development, MolPort ID, and IUPAC name. This supplementary figure refers to main Figure 7.*

A.

Paper name	No.	Activity*	SMILES	MolPort ID	IUPAC Name
MyA		***	CC1C(=O)C(C(=O)C(C1=O)(C)C)C(=O)CCC2=CC=CC=C2		2,2,4-trimethyl-6-(3-phenylpropanoyl)cyclohexane-1,3,5-trione
ETHi-74-12-1	1	-	COc1ccc(C=CC(=O)C2C(=O)OC(C)=CC2=O)cc1OC	044-402-240	3-[3-(3,4-dimethoxyphenyl)prop-2-enoyl]-6-methyl-3,4-dihydro-2H-pyran-2,4-dione
ETHi-74-12-2	2	**	CC1=CC(=O)C(C(=O)C=Cc2ccccc2C)C(=O)O1	044-250-811	6-methyl-3-[3-(2-methylphenyl)prop-2-enoyl]-3,4-dihydro-2H-pyran-2,4-dione
ETHi-68-6-3	3	-	O=C(\C=C\c1ccccc1)C1C(=O)c2ccccc2C1=O	002-577-535	2-[(2E)-3-phenylprop-2-enoyl]-2,3-dihydro-1H-indene-1,3-dione
ETHi-84-16-4	4	***	CC1(C)CC(=O)C(C(=O)Cc2ccccc2)C(=O)C1	002-137-086	5,5-dimethyl-2-(2-phenylacetyl)cyclohexane-1,3-dione
ETHi-68-6-5	5	-	Cc1ccc(\C=C\C(=O)C2C(=O)c3ccccc3C2=O)cc1	002-161-837	2-[(2E)-3-(4-methylphenyl)prop-2-enoyl]-2,3-dihydro-1H-indene-1,3-dione
ETHi-68-6-6	6	-	O=C1C(=Cc2ccccc2)C(=O)c2ccccc12	000-489-111	2-(phenylmethylidene)-2,3-dihydro-1H-indene-1,3-dione
ETHi-68-6-3	7	**	CC1(C)CC(=O)C(C(=O)c2ccccc2)C(=O)C1	001-957-279	2-benzoyl-5,5-dimethylcyclohexane-1,3-dione
ETHi-0-0-28	28	-	CC1(CCCCC1)C(=O)CCc1ccccc1	016-631-668	1-(1-methylcyclohexyl)-3-phenylpropan-1-one
ETHi-74-12-9	9	-	CCOc1cc(C=CC(=O)C2C(=O)OC(C)=CC2=O)ccc1OC	044-251-050	3-[3-(3-ethoxy-4-methoxyphenyl)prop-2-enoyl]-6-methyl-3,4-dihydro-2H-pyran-2,4-dione
ETHi-74-12-10	10	**	CC1=CC(=O)C(C(=O)C=Cc2ccc(cc2)C(C)(C)C)C(=O)O1	044-250-863	3-[3-(4-tert-butylphenyl)prop-2-enoyl]-6-methyl-3,4-dihydro-2H-pyran-2,4-dione
ETHi-68-6-11	11	**	O=C(\C=C\c1ccco1)C1C(=O)c2ccccc2C1=O	001-917-147	2-[(2E)-3-(furan-2-yl)prop-2-enoyl]-2,3-dihydro-1H-indene-1,3-dione
ETHi-74-12-12	12	***	Cc1cc(O)c(C(=O)\C=C\c2ccccc2OC(F)F)c(=O)o1	002-759-778	3-[(2E)-3-[2-(difluoromethoxy)phenyl]prop-2-enoyl]-4-hydroxy-6-methyl-2H-pyran-2-one
ETHi-0-0-13	13	-	Cc1ccc(cc1)C(=O)CCC1CCCCC1=O	000-805-492	2-[3-(4-methylphenyl)-3-oxopropyl]cyclohexan-1-one
ETHi-74-12-14	14	***	CCc1ccc(C=CC(=O)C2C(=O)OC(C)=CC2=O)cc1	044-250-851	3-[3-(4-ethylphenyl)prop-2-enoyl]-6-methyl-3,4-dihydro-2H-pyran-2,4-dione
ETHi-74-12-15	15	***	CC1=CC(=O)C(C(=O)C=Cc2cccc(Cl)c2Cl)C(=O)O1	044-250-754	3-[3-(2,3-dichlorophenyl)prop-2-enoyl]-6-methyl-3,4-dihydro-2H-pyran-2,4-dione
ETHi-74-12-16	16	***	Cc1cc(O)c(C(=O)\C=C\c2ccc(F)cc2)c(=O)o1	005-287-602	3-[(2E)-3-(4-fluorophenyl)prop-2-enoyl]-4-hydroxy-6-methyl-2H-pyran-2-one
ETHi-74-12-17	17	**	CN(C)c1ccc(C=CC(=O)C2C(=O)OC(C)=CC2=O)cc1	044-250-746	3-{3-[4-(dimethylamino)phenyl]prop-2-enoyl}-6-methyl-3,4-dihydro-2H-pyran-2,4-dione
ETHi-74-12-18	18	**	CC1=CC(=O)C(C(=O)C=Cc2cc(C)ccc2C)C(=O)O1	044-207-442	3-[3-(2,5-dimethylphenyl)prop-2-enoyl]-6-methyl-3,4-dihydro-2H-pyran-2,4-dione
ETHi-0-0-19	19	**	O=C(CCC1CCCC1=O)c1ccccc1	001-782-962	2-(3-oxo-3-phenylpropyl)cyclopentan-1-one
ETHi-74-12-20	20	**	CC1=CC(=O)C(C(=O)C=Cc2cccc(Oc3ccc(Cl)cc3)c2)C(=O)O1	044-250-673	3-{3-[3-(4-chlorophenoxy)phenyl]prop-2-enoyl}-6-methyl-3,4-dihydro-2H-pyran-2,4-dione
ETHi-74-12-21	21	-	CC1=CC(=O)C(C(=O)C=Cc2cccc(C)c2)C(=O)O1	044-250-849	6-methyl-3-[3-(3-methylphenyl)prop-2-enoyl]-3,4-dihydro-2H-pyran-2,4-dione
ETHi-74-12-22	22	***	CC1=CC(=O)C(C(=O)C=Cc2cccc(F)c2)C(=O)O1	044-250-804	3-[3-(3-fluorophenyl)prop-2-enoyl]-6-methyl-3,4-dihydro-2H-pyran-2,4-dione
ETHi-74-12-23	23	***	Cc1cc(O)c(C(=O)\C=C\c2ccc(OC(F)F)cc2)c(=O)o1	017-264-427	3-[(2E)-3-[4-(difluoromethoxy)phenyl]prop-2-enoyl]-4-hydroxy-6-methyl-2H-pyran-2-one
ETHi-74-12-24	24	-	CC1(C)CC(=O)C(Cc2ccccc2)C(=O)C1	004-947-021	2-benzyl-5,5-dimethylcyclohexane-1,3-dione
ETHi-4-12-25	25	-	CC1=CC(=O)C(C(=O)\C=C\c2ccc(cc2)[N+]([O-])=O)C(=O)O1	046-693-572	6-methyl-3-[(2E)-3-(4-nitrophenyl)prop-2-enoyl]-3,4-dihydro-2H-pyran-2,4-dione
ETHi-74-12-26	26	-	CC1=CC(=O)C(C(=O)\C=C\c2ccccc2)C(=O)O1	003-285-479	6-methyl-3-[(2E)-3-phenylprop-2-enoyl]-3,4-dihydro-2H-pyran-2,4-dione
ETHi-68-6-27	27	-	O=C1\C(Cc2ccccc12)=C\c1ccccc1	001-835-266	(2E)-2-(phenylmethylidene)-2,3-dihydro-1H-inden-1-one

B.

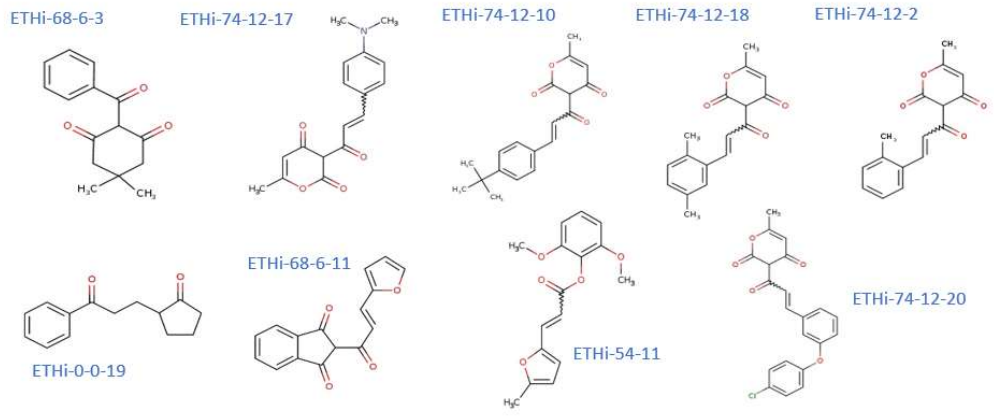

C.

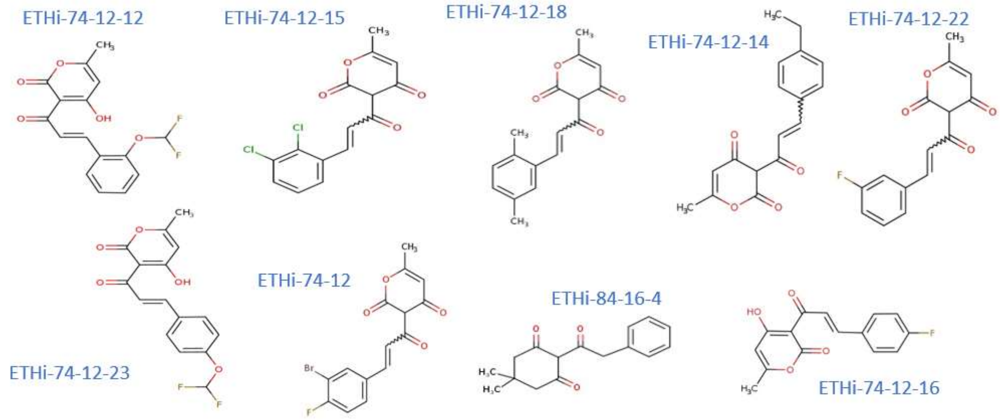

Supplementary Figure S14: *Second round of bioactivity analysis of novel ethylene inhibitors (ETHi) for efficacy in reducing the phenotype identified in blocking ethylene production in D. discoideum. A. Compounds identified in the first round of testing (Sup. Fig. 13) were used to identify related compounds, that were tested for developmental delay effects in D. discoideum, to*

*mimic that shown for established ethylene inhibitors and MyA, blocking development at the mound stage, where ‘***’ = delayed to mounds (MyA-like) , ‘**’ = mostly mounds several breakthrough, ‘*’ = less delayed development and ‘-’ = no effect on development. Compounds are listed as preliminary names, activity related to inhibition of development, MolPort ID, and IUPAC name. B. and C. Compound structures are indicated for highly potent and intermediary potency respectively. This supplementary figure refers to main Figure 7.*

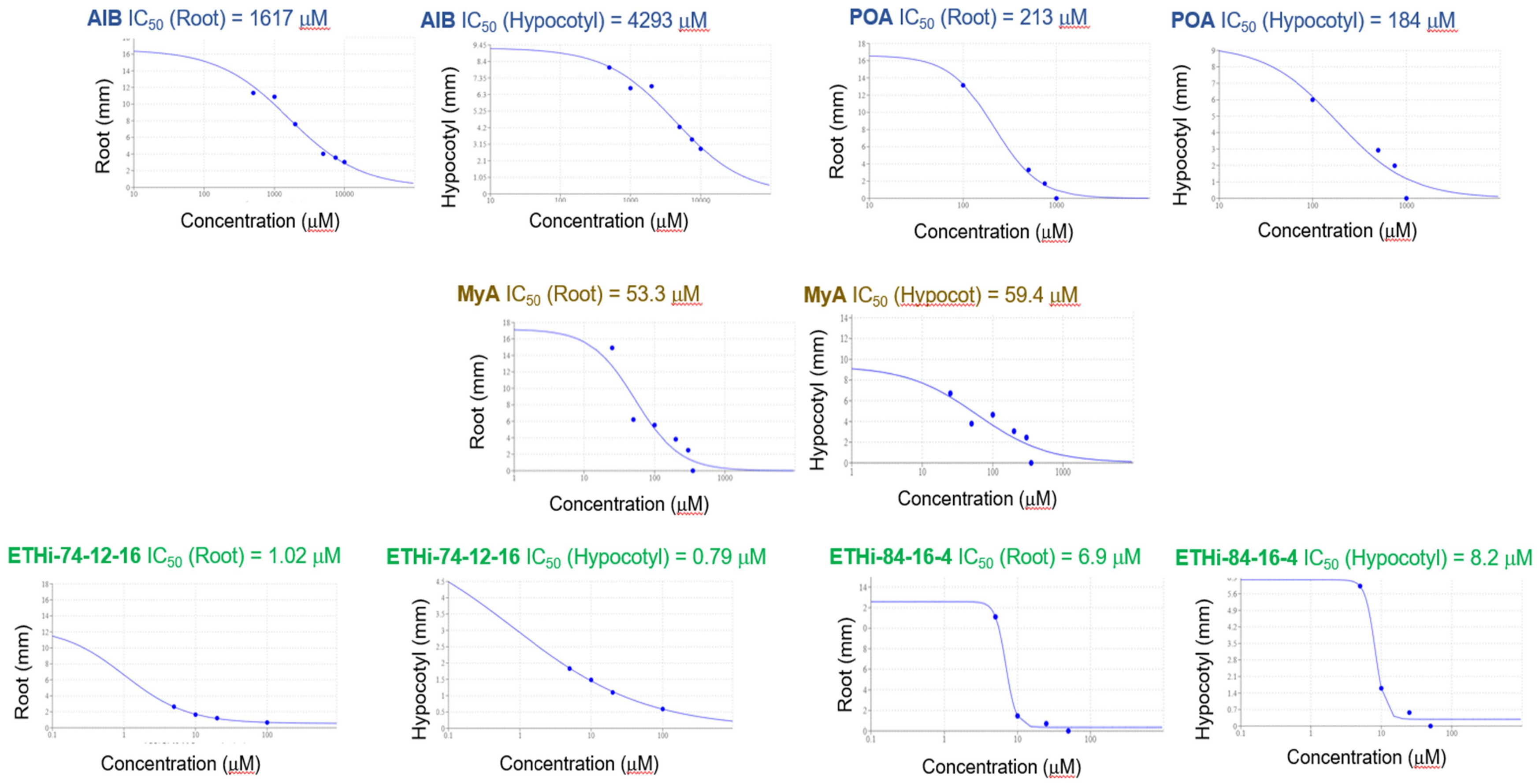

Supplementary Figure S15: *Comparison root and hypocotyl extension inhibition of established ACO inhibitors, MyA and novel compounds in A. thaliana seedlings. This supplementary figure refers to main Figure 8.*
